# Development and validation of interpretable multimodal clinical-radiomics models for predicting epileptogenic foci and surgical outcomes in tuberous sclerosis complex: A multicenter study

**DOI:** 10.1371/journal.pdig.0001259

**Published:** 2026-02-26

**Authors:** Yang Li, Xingye Liu, Jie Li, Mingxun Xie, Shujing Li, Kaixuan Huang, Jiaxi Zhao, Xin Chen, Zeng He, Jing He, Limeng Sun, Renrong Jiang, Chun Cui, Li Wang, Zhonghong Liu, Song Zhang, Haifeng Shu, Shengqing Lv, Chunqing Zhang, Dong Zhang, Di Wang, Hui Yang, Qiang Guo, Hongping Tan, Xiaolin Yang, Shiyong Liu, Zhongke Wang

**Affiliations:** 1 Comprehensive Epilepsy Center, Department of Neurosurgery, Xinqiao Hospital, Army Medical University, Chongqing, China; 2 Department of Radiology, Xinqiao Hospital, Army Medical University, Chongqing, China; 3 Department of Neurosurgery, The General Hospital of Western Theater Command, Sichuan, China; 4 Chongqing Institute for Brain and Intelligence, Guangyang Bay Laboratory, Chongqing, China; 5 Department of Neurosurgery, Armed Police Hospital of Chongqing, Chongqing, China; 6 Department of Nuclear Medicine, Xinqiao Hospital, Army Medical University, Chongqing, China; 7 Department of Neurology, Xinqiao Hospital, Army Medical University, Chongqing, China; 8 School of Information Science and Engineering, Chongqing Jiaotong University, Chongqing, China; 9 Epilepsy Center, Guangdong 999 Brain Hospital, Affiliated Brain Hospital of Jinan University, Guangdong, China; Xiangtan Central Hospital, CHINA

## Abstract

Precise localization and resection of epileptogenic (epi) foci from multiple cortical foci determine surgical outcomes in the tuberous sclerosis complex (TSC). Although the use of intracranial electroencephalography (EEG) for detecting epileptic discharges remains the gold standard for identifying epi foci, its invasiveness and cost limit clinical application. We aimed to develop and validate a noninvasive, clinically applicable predictive model for epi foci identification and surgical outcome assessment in patients with TSC. This multicenter study focused on three retrospective cohorts and one prospective cohort from three comprehensive epilepsy centers from June 2013 to October 2024. Comprehensive clinical and imaging data (CT, MRI, and ^18^F-FDG PET) of cortical foci were collected. Nineteen individual machine learning (ML) models and three ensemble ML models (voting, averaging and super-learner [SL]) were developed on the basis of the clinical and radiomics features of cortical foci. Model performance was evaluated by using the area under the curve (AUC), accuracy, precision, specificity, and sensitivity values, along with the F1 score, with additional validation being conducted via decision curve analysis (DCA) and calibration curves. Follow-up data were collected at 1, 3, and >5 years to validate the ability of the ML models to predict long-term postoperative outcomes. Non-epi foci were clustered by using the k-means algorithm to investigate the mechanisms underlying postoperative epileptogenic transformation. A web-based tool was developed to provide a user-friendly interface for clinical application. A total of 665 cortical foci (epi foci, n = 161; non-epi foci, n = 504) were included in this study. The model integrating multimodal clinical-radiomics features performed better than the individual models based only on single-modal clinical or radiomics features did. The ensemble SL model using clinical-radiomics features demonstrated the best stability and superior predictive performance compared to those of individual models and an additional two ensemble models in prospective (AUC: 0.92) and two retrospective cohorts (AUCs: 0.91 and 0.87); moreover, it outperformed previously reported prediction models. In addition, the SL model effectively predicted 1-, 3- and >5-year surgical outcomes (AUCs: 0.93, 0.91, and 0.92, respectively). K-means revealed two clusters of non-epi foci, including those foci with epileptogenic potential and those without, which were potentially confirmed by the follow-up data. The web-based tool significantly increased the accuracy of junior clinicians (from 0.61 to 0.78), which matched the accuracy of senior clinicians (0.80). The multimodal clinical-radiomics model represents a noninvasive tool for predicting epi foci, guiding preoperative evaluation, addressing diagnostic discrepancies and enabling personalized treatment strategies in patients with TSC. The clinical application of artificial intelligence (AI)-driven clinical-radiomics models provides a useful tool and auxiliary reference for clinicians in preoperative epileptogenic foci prediction.

## Author summary

In this multicenter study, we developed and validated noninvasive predictive models for epi foci localization and surgical outcomes in patients with TSC by integrating multimodal clinical-radiomics features from three retrospective cohorts and one prospective cohort from June 2013 to October 2024. Nineteen individual and 3 ensemble machine learning (ML) models were constructed, with the ensemble super-learner (SL) model demonstrating superior performance in prospective (AUC: 0.92) and two retrospective cohorts (AUCs: 0.91 and 0.87); additionally, it outperformed other reported prediction models. The clinical-radiomics SL model predicted the 1-, 3- and >5-year surgical outcomes (AUCs: 0.93, 0.91, and 0.92, respectively) of cortical foci. Furthermore, a web-based tool of predictive models was developed, and it significantly increased the accuracy of junior clinicians (from 0.61 to 0.78), which matched that of senior clinicians (0.80). The clinical-radiomics SL model represents a promising tool to predict epi foci, address diagnostic discrepancies among clinicians of varying levels of expertise, and enable personalized treatment strategies while minimizing the invasiveness and financial strain of TSC patients. Due to its robust performance, this model demonstrates considerable translational potential in both TSC patients and all epilepsy patients with multiple epi foci. The clinical application of this model provides an AI-driven tool and auxiliary reference for clinicians in preoperative epileptogenic foci prediction.

## Introduction

Tuberous sclerosis complex (TSC) is a multisystem, autosomal dominant syndrome affecting approximately 1 in 6,000 live births. Epilepsy, which occurs in 80–90% of patients with TSC, is the most common neurological manifestation of this disease, with nearly two-thirds of these patients developing drug-resistant epilepsy (DRE) [1,2]. As reported in the 2021 updated international diagnostic criteria for TSC [2], “multiple cortical tubers” in an individual patient are a characteristic feature of TSC, and it has been used to replace cortical dysplasia in diagnostic criteria, thus suggesting the pivotal role of multiple cortical foci in the epileptogenesis of TSC. Previous studies have demonstrated that nearly all TSC patients exhibit multiple cortical tubers, which serve as potential seizure-onset zones [3]. Our previous nationwide multicenter retrospective study of resective epilepsy surgery for TSC showed that 92% of patients had ≥ 4 cortical tubers, whereas only 9 (2.3%) among the 383 enrolled patients had a single cortical tuber. Importantly, not all cortical tubers are origins of epileptic seizures, and postoperative outcomes have confirmed that only a subset of the tubers per patient are epileptogenic (epi) foci [3]. Thus, the key to the preoperative evaluation of TSC patients is the precise identification of epi foci from multiple potential seizure-onset zones. In contrast, for other types of malformation of cortical development (MCD), such as focal cortical dysplasia (FCD), epi foci typically represent the identified structural lesion and rarely present with multiple independent potential seizure-onset zones [4,5]. Given this distinctive pathology, TSC represents a suitable model for investigating epi foci among multiple potential seizure-onset zones due to the common presence of both epi and non-epi foci within the same individual.

Our previous nationwide multicenter study reported that resection of epi foci is the most effective intervention for intractable epilepsy in TSC patients [3]. The precise preoperative localization of epi foci among multiple cortical tubers is crucial for determining resection strategies, surgical outcomes, and prognosis in TSC-related epilepsy, yet remains a significant clinical challenge [6]. Although intracranial electroencephalography (EEG) remains the gold standard for the identification of epi foci, its invasive nature and expense limit its clinical application. Noninvasive neuroimaging methods, including computed tomography (CT), magnetic resonance imaging (MRI), and ^18^F-fluorodeoxyglucose positron emission tomography (^18^F-FDG PET), are increasingly being used as imaging biomarkers to predict epi foci. The combination of multimodal features of MRI with PET has proven to be valuable in detecting temporal lobe epilepsy with dual pathology [7]. Advances in neuroimaging are crucial for identifying cortical malformations that cause seizure disorders. Neuroimaging gradient alterations in FCD II can be used to guide a suitable resection range and predict postoperative seizure outcomes [8]. Additionally, characteristic TSC lesions on early brain MRI are correlated with seizure development and neurodevelopmental outcomes during the first 2 years of life [9]. Neuroimaging studies have significantly contributed to lesion detection by revealing group-level differences compared with healthy controls; however, their clinical translatability is limited due to insufficient individual-level precision.

Machine learning (ML) algorithms represent highly suitable for individualized medicine due to the fact that they can be trained with vast amounts of data and are able to consider novel data sources, including genetic profiles, imaging, EEG recordings, and physiological data [10,11]. ML models based on brain images can predict epilepsy risk in several neurological disorders, along with classifying epilepsy patients with high accuracy and identifying neuroanatomical features associated with epilepsy, even in non-lesional (MRI-negative) cases [12–15]. Radiomics is described as a highly automated computational method that can extract and analyze large amounts of advanced quantitative imaging features [16]. Its utility has been demonstrated in predicting postoperative seizure recurrence [17], poststroke cognitive impairment [18], and hematoma expansion after intracerebral hemorrhage (ICH) [19]. Although radiomics and ML algorithms have been applied to identify epi foci in intractable epilepsy [20], their potential use for predicting such foci in TSC remains unexplored.

In this study, we developed and validated a predictive ML model integrating multimodal clinical and radiomics features extracted from medical records, as well as CT, MRI, and ^18^F-FDG PET images of 665 cortical foci in retrospective and prospective cohorts. We aimed to predict epi foci from multiple cortical foci and long-term surgical outcomes in patients with TSC. Importantly, this study introduces an optimal noninvasive method that may serve as an alternative to intracranial EEG, thereby potentially mitigating intracranial injury risks and reducing healthcare burdens for patients with TSC.

## Materials and methods

### Study cohort

**Fig 1:**
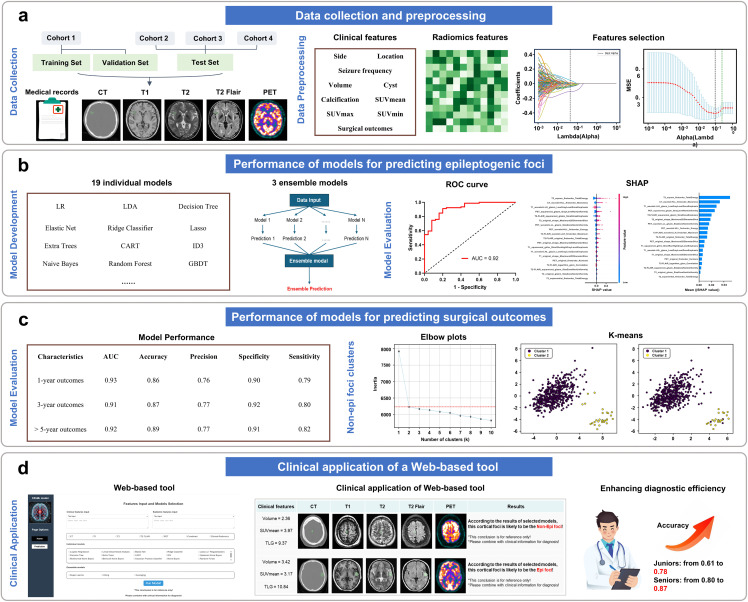
The overall framework of the study. **(a)** Data collection and preprocessing. **(b)** Performance of models for predicting epileptogenic foci. **(c)** Performance of models for predicting surgical outcomes. **(d)** Clinical application of a Web-based tool.

**Fig 2:**
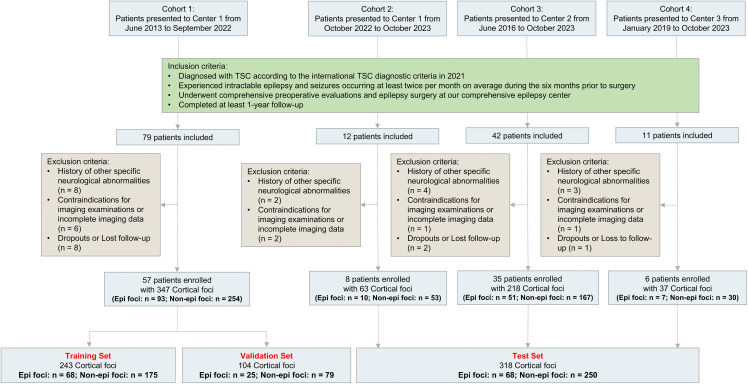
Flowchart of patient inclusion and exclusion. TSC, tuberous sclerosis complex; Epi foci, epileptogenic foci; Non-epi foci, Non-epileptogenic foci.

The overall framework of this study is illustrated in Fig 1 and S1 Text. All patients who underwent resection surgery were enrolled after they had presented to the three epilepsy centers before October 2023, with at least one year of follow-up in October 2024. Presurgical evaluation and surgery adhered to the criteria for pediatric epilepsy surgery center levels of care [21]. This study involved a retrospective cohort to train and validate the models (cohort 1) and a prospective cohort to test the models (cohort 2) from the comprehensive epilepsy center of Xinqiao Hospital, as well as two retrospective cohorts to test the models from the comprehensive epilepsy center of Guangdong 999 Brain Hospital (cohort 3) and General Hospital of Western Theater Command (cohort 4). The inclusion criteria included (i) diagnosed with TSC according to the international TSC diagnostic criteria in 2021 [2]; (ii) experienced intractable epilepsy and seizures at least twice per month on average during the six months prior to surgery; (iii) underwent comprehensive preoperative evaluations and epilepsy surgery at the comprehensive epilepsy center; and (iv) completed at least 1 year of follow-up. The exclusion criteria included (i) a history of other specific neurological abnormalities, mainly including encephalitis, hydrocephalus, intracerebral hemorrhage or cerebrovascular disease; (ii) contraindications for imaging examinations or incomplete imaging data; and (iii) dropout or loss to follow-up. A total of 144 patients from four cohorts were considered for inclusion, among whom 17 patients had a history of other specific neurological abnormalities, 10 patients had contraindications for imaging examinations or incomplete imaging data, and 11 patients dropped out or were lost to follow-up; these patients were ultimately excluded. The results are reported in accordance with the Standards for Reporting Diagnostic Accuracy Studies (STARD) guidelines (S1 and S2 Appendices). The detailed criteria are shown in Fig 2.

### Clinical feature acquisition

The clinical characteristics of the patients, including patient age, sex, type of gene mutation, seizure onset, seizure frequency, numbers of anti-seizure medications (ASMs) and cortical foci, results of ictal epileptic discharge, surgical approach and surgical outcomes, were obtained from medical records and are shown in S1 Table. The surgical outcomes were assessed by using the International League Against Epilepsy (ILAE) classification, and the surgical outcomes were dichotomized into seizure freedom (SF; ILAE I) and no-seizure freedom (No-SF; ILAE II–VI) categories according to previous studies [22,23]. This approach was primarily intended to enhance the statistical power of the predictive models and focus on the clinical distinction between epi and non-epi foci.

Comprehensive preoperative examinations were performed. First, noninvasive methods were used to identify epi foci [3]. Both the literature and our previous studies consistently showed that larger, calcified cortical foci were associated with greater epileptogenic potential in patients with TSC [24,25]. Our previous nationwide multicenter retrospective study on resective epilepsy surgery in TSC patients defined “outstanding cortical foci” as those > 3–4 cm with a nidus of calcification and demonstrated that the presence of such outstanding foci influences postoperative seizure freedom at 1-, 4-, and 10-year follow-ups; moreover, seizure freedom was often achieved in patients who underwent resective epilepsy surgery targeting an outstanding focus [3]. Another prospective nationwide multicenter cohort study investigating the effectiveness and safety of resective surgery for TSC also defined outstanding cortical foci as those >3–4 cm in size, with relatively clear boundaries and evidence of calcification and/or cystic changes [26]. In this study, epi foci were defined as (i) cortical foci with focal ictal and interictal epileptiform discharges in the same brain region on scalp EEG with consistent seizure symptoms; (ii) outstanding cortical foci with focal ictal or interictal epileptiform discharges in the same brain region on scalp EEG with consistent seizure symptoms; or (iii) outstanding cortical foci with focal ictal and interictal epileptiform discharges in the same brain region on scalp EEG. For patients with multiple epi foci or discrepancies among clinical symptoms, imaging results and electrophysiology findings, intracranial electrodes were invasively implanted to record electrical activity and identify epi foci. Clinical features of the cortical foci are shown in Table 1, including foci side, location, seizure frequency, volume, cyst, calcification, mean standardized uptake value (SUVmean), SUVmax, SUVmin, total lesion glycolysis (TLG) and surgical outcomes. The SUV is calculated as [tissue radioactivity concentration (kBq/ml)]/[(injected dose (MBq))/body weight (kg)].

**Table 1:** Clinical characteristics of Epi and Non-Epi foci in four cohorts.

Clinical features	Cohort 1			Cohort 2			Cohort 3			Cohort 4		
	Epi foci (n = 93)	Non-Epi foci (n = 254)	*p* Value	Epi foci (n = 10)	Non-Epi foci (n = 53)	*p* Value	Epi foci (n = 51)	Non-Epi foci (n = 167)	*p* Value	Epi foci (n = 7)	Non-Epi foci (n = 30)	*p* Value
**Side**												
Right	58(62.4%)	136(53.5%)		6(60.0%)	30(56.6%)		34(66.7%)	90(53.9%)		5(71.4%)	15(50.0%)	
Left	35(37.6%)	118(46.5%)		4(40.0%)	23(43.4%)		17(33.3%)	77(46.1%)		2(28.6%)	15(50.0%)	
**Location**												
Frontal	38(40.9%)	105(41.3%)		4(40.0%)	14(26.4%)		14(27.5%)	54(32.3%)		2(28.6%)	11(36.7%)	
Temporal	12(12.9%)	34(13.4%)		1(10.0%)	5(9.4%)		5(9.8%)	13(7.8%)		1(14.3%)	4(13.3%)	
Parietal	25(26.9%)	68(26.8%)		2(20.0%)	20(37.7%)		19(37.3%)	55(32.9%)		2(28.6%)	7(23.3%)	
Occipital	14(15.1%)	39(15.4%)		2(20.0%)	12(22.6%)		13(25.5%)	41(24.6%)		2(28.6%)	5(16.7%)	
Insular	4(4.3%)	8(3.1%)		1(10.0%)	2(3.8%)		—	4(2.4%)		—	3(10.0%)	
**Seizure frequency**												
Daily	49(52.7%)	—		5(50.0%)	—		27(52.9%)	—		5(71.4%)	—	
Weekly	21(22.6%)	—		3(30.0%)	—		3(5.9%)	—		2(28.6%)	—	
Monthly	18(19.4%)	—		2(20.0%)	—		21(41.2%)	—		—	—	
Yearly	5(5.4%)	—		—	—		—	—		—	—	
**Volume**	3.67	2.73	p < 0.01	4.07	2.94	p < 0.01	4.1	2.85	P < 0.01	3.76	3.02	P < 0.01
**Cyst**	18(19.4%)	41(16.1%)		1(10.0%)	9(17.0%)		14(27.5%)	28(16.8%)		1(14.3%)	6(20.0%)	
**Calcification**	36(38.7%)	102(40.2%)		3(30.0%)	17(32.1%)		12(23.5%)	56(33.5%)		2(28.6%)	11(36.7%)	
**SUVmean**	3.02	4.13	p < 0.05	2.95	4.37	p < 0.05	3.07	4.34	P < 0.05	2.77	4.21	P < 0.01
**SUVmax**	5.19	6.43	p < 0.05	5.37	6.52		5.26	7.04	P < 0.05	4.92	6.28	
**SUVmin**	2.24	3.15		2.41	3.18		2.12	3.22		2.23	3.44	
**TLG**	11.24	12.38	p < 0.01	11.92	13.11	p < 0.01	11.77	12.59	P < 0.01	10.97	12.76	P < 0.05

Epi foci, epileptogenic foci; Non-Epi foci, Non-epileptogenic foci; SUV, standard uptake value; TLG, total lesion glycolysis**.**

### Imaging data collection and processing

All of the enrolled patients underwent CT, MRI and ^18^F-FDG PET scans. The detailed scanner modalities, vendors and parameters from the three centers are shown in the S2 Table. Briefly, MRI scans were conducted by using a 3.0-T system, including T1-weighted, T2-weighted, and fluid-attenuated inversion recovery (T2 FLAIR) sequences. For ^18^F-FDG PET scans, patients fasted for ≥ 4 h before scanning, had a blood glucose concentration < 11.1 mmol/L at the time of FDG injection, and underwent PET acquisition at 1 h postinjection of 3 MBq/kg FDG. Attenuation correction was performed by using a 64-slice multidetector-row spiral CT scanner. All of the patients were awake and in a resting state; moreover, no patients experienced seizures during the imaging scans.

The images were aligned with the standard space, and standard pediatric magnetic resonance brain images were used as references. The spatial normalization process involved the utilization of the normalization toolbox within the SPM12 software and the implementation of the DARTEL algorithm. This algorithm is used to establish a mapping relationship between patient images and standard pediatric brain images, thereby transforming the spatial positions of patient images to align them with those of standard brain images. Additionally, motion and field bias corrections were performed during the normalization process to ensure the accuracy (Acc) and reliability of the final results. Ultimately, this process facilitates the alignment of images acquired from the same patient at different time points or modalities into a consistent standard space position. All of the multimodal images (CT, T1, T2, and ^18^F-FDG PET) were subsequently rigidly co-registered to each subject’s native T2 FLAIR image because the cortical foci were clearly defined on this sequence [27]. The T2 FLAIR image was normalized to the DARTEL-derived standard template. This approach ensured that all of the modalities were accurately aligned and spatially normalized to the same MNI standard space. Regions of interest (ROIs) were independently delineated on the T2 FLAIR images by two senior neuroradiologists, each with 10 years of clinical experience in brain neuroimaging interpretation. Both neuroradiologists were fully blinded to the patients’ clinical information and pathological results throughout the delineation process. The ROIs were also applied to the coregistered CT, T1, T2, T2-FLAIR and ^18^F-FDG PET images.

### Radiomics feature extraction

Radiomics features, including the first-order, shape, gray-level co-occurrence matrix (GLCM), gray-level run length matrix (GLRLM), gray-level size zone matrix (GLSZM), neighborhood gray-tone difference matrix (NGTDM), and GLCM feature families, were extracted by using Python 3.12 along with the PyRadiomics library. These descriptors individually capture aspects of the image intensity distribution, morphology, texture, and interpixel relationships. To ensure the repeatability and robustness of these features, uniform parameter configurations were applied across each modality, encompassing the grayscale levels, orientations, and distances that are utilized in texture analysis. The intra-reader and inter-reader intraclass correlation coefficients (ICCs) were performed according to previous studies [28,29]. The intra–reader test–retest ICC was determined from the readings of one of the senior neuroradiologists, who independently delineated all the ROIs twice, with a 2-week interval between the two sessions. During the second delineation, he was blinded to his previous results and all patients’ information to avoid recall bias. For each radiomics feature that was extracted from the ROIs, the intra-reader ICC values were calculated. The results demonstrated ICC values ranging from 0.82 to 0.97, which were well above the 0.75 threshold necessary for acceptable reproducibility. This result confirms the high consistency in the radiologist’s ROI delineation over time. Inter-reader validation was further conducted and involved two senior neuroradiologist, who independently delineated all of the ROIs and were also blinded to the patients’ clinical data. Inter-reader ICC values were calculated by comparing radiomics feature values from the delineations of the two radiologists. The inter-reader ICCs ranged from 0.79 to 0.95, exceeding the 0.75 threshold and confirming reliable consistency across independent readers. For the models based on the single-modal imaging data of CT, T1, T2, T2 FLAIR and ^18^F-FDG PET, LASSO was separately applied to the radiomics features of each imaging modality. For the models based on the multimodal imaging data, LASSO was applied to the combined radiomics features. Additionally, 10-fold cross validation was used to choose the optimal lambda of the LASSO model via minimum criteria. The lambda with the minimum mean squared error serves as the criterion for selecting predictive variables [30,31].

### Model development and validation

Nineteen individual ML models were constructed based on the radiomics features, which included almost all types of commonly used models, such as logistic regression (LR), linear discriminant analysis (LDA), decision tree (DT), Gaussian naive Bayes (GNB), and random forest (RF) models. The individual models were trained on the training set from cohort 1, and their hyperparameters were tuned by using the validation set from cohort 1. After hyperparameter tuning, each individual model’s performance was evaluated by using the area under the curve (AUC) values on the validation set. Based on these AUC values of each individual model in the validation set, the top five individual models were selected and combined to develop the ensemble models (including voting, averaging and super-learner [SL] models). The ensemble models were retrained with the training set from cohort 1 and validated with the validation set from cohort 1. Furthermore, all of the individual models and ensemble models were tested by using the test set from cohorts 2–4. All of the models can be implemented by using the Python packages (S3 Appendix) and are easy to interpret in clinical practice.

The AUC, 95% CI, accuracy, precision, specificity, and sensitivity values, as well as the F1 score, were used to evaluate the model performance. The Shapley Additive Explanations (SHAP) algorithm was used to visualize the selected important features. The SHAP values were calculated to determine the individual contributions of the selected radiomics features to the model’s prediction and outcomes. Summary plots and bar charts were constructed to demonstrate the importance of each selected radiomics feature and the clinical practicability of the model. Additionally, decision curve analysis (DCA) was performed to evaluate the net benefit of distinguishing clinical epi foci. Calibration curves were constructed to assess the calibration of the ML models by comparing the observed outcomes with the predicted probabilities, thereby providing an intuitive method to evaluate the alignment between a model’s predictions and actual observations, as well as to evaluate the accuracy and reliability of the predictive models.

The 1-, 3- and > 5-year surgical outcomes were followed, and the predictive performance of long-term surgical outcomes in the ML models were evaluated. K-means algorithm analysis was employed to classify non-epi foci into distinct clusters based on clinical and radiomics features, aiming to explore the mechanisms underlying the ability of the ML models to predict long-term postoperative outcomes. The optimal number of clusters was visually determined via elbow plots.

### Web-based tool application

The web-based tool of the model was created to facilitate the clinical application of the model by using the Python programming language and Flask framework. The web-based tool had been packaged into a Docker image, which is publicly available on Docker Hub at the following repository: crmloftsc/web-based_tool:v1.0. Users can access and run the tool by simply executing the command docker pull crmloftsc/web-based_tool:v1.0 to download the image, followed by docker run -p 5000:5000 crmloftsc/web-based_tool:v1.0 to start the container. Once the container is running, the web tool will be accessible in the browser at the address http://localhost:5000. This web-based tool can assist clinicians in predicting the epileptogenicity of cortical foci based on its clinical and radiomics features. Three junior clinicians (5 years of clinical practice) and three senior clinicians (10 years of clinical practice) who were unaware of the epileptogenicity of cortical foci participated in the application of the web-based tool.

### Statistical analysis

Statistical analysis was performed by using Python (version 3.12). Cortical foci were categorized as epi or non-epi foci based on the comprehensive evaluation of clinical symptoms, imaging findings, and electrophysiological data, as detailed in the assessment of clinical features section of the Methods section. The Levene’s test was used to determine the homogeneity of variance. Continuous variables exhibiting homogeneity of variance were compared by using unpaired two-tailed *t* tests, and continuous variables with uneven variances were compared by using Welch’s *t* tests. Quantitative data are presented as the mean values. For predictive validation, receiver operating characteristic curves were constructed with 95% CIs, and the AUROC was used to evaluate model performance. Additionally, to comprehensively quantify model performance, standard metrics of accuracy, precision, specificity, sensitivity and F1 score were computed. Statistical significance was set at *P* < 0.05.

### Role of the funding source

The funders played no role in study design, data collection, data analyses, interpretation, or writing of report. The sole responsibility for the content of this publication lies with the authors.

### Ethics

All procedures were performed according to the guidelines of the Declaration of Helsinki of the World Medical Association and the Ethics Committee guidelines of Xinqiao Hospital (approval number: RN202502601), and this study had been registered on Chinese Clinical Trial Registry with the registered number of ChiCTR2500098144. All patients signed informed consent forms.

## Results

### Clinical characteristics of patients and cortical foci

A total of 665 cortical foci (161 epi and 504 non-epi foci) from four cohorts were ultimately collected, including 347 cortical foci (93 epi and 254 non-epi foci) from 57 patients retrospectively enrolled in cohort 1, 63 cortical foci (10 epi and 53 non-epi foci) from 8 patients prospectively enrolled in cohort 2, 218 cortical foci (51 epi and 167 non-epi foci) from 35 patients retrospectively enrolled in cohort 3, and 37 cortical foci (7 epi and 30 non-epi foci) from 6 patients retrospectively enrolled in cohort 4. All of the patients underwent epilepsy surgery and had complete medical records, imaging data, and at least 1 year of follow-up data (Fig 2). The dataset of cohort 1 was randomly divided into a training set (70%) and a validation set (30%), thereby ensuring that there were no significant differences in baseline demographics. All the cortical tubers from a single patient were completely assigned to either the training or validation set and that no cortical tubers from the same patient were divided between the two sets. This approach fundamentally prevents data leakage arising from within-patient tuber clustering. The datasets of cohorts 2, 3 and 4 were used as the test set. There was no difference observed in the demographics of the patients among the four cohorts (S1 Table). In terms of the clinical features of cortical foci, the epi foci exhibited a greater volume and lower SUVmean and TLG than the non-epi foci in both cohorts; moreover, the epi foci exhibited lower SUVmax values in cohorts 1 and 3 (Table 1). Therefore, volume, SUVmean, and TLG, which demonstrated notable differences across both cohorts, were selected for the development of the ML models.

### Performance of the radiomics models for predicting epileptogenic foci

A total of 1316 radiomics features were extracted from each of the cortical foci on the single-modal imaging data of CT, T1, T2, T2 FLAIR and ^18^F-FDG PET. The multimodal imaging data included 6580 radiomics features (5 x 1316) from combined CT, T1, T2, T2 FLAIR and ^18^F-FDG PET images. After reproducibility was assessed by using the ICCs and features were selected by using LASSO, three optimal radiomics features were identified for the CT model, six optimal radiomics features were identified for the T1 model, five optimal radiomics features were identified for the T2 model, four optimal radiomics features were identified for the T2 FLAIR model, seven optimal radiomics features were identified for the PET model, and nineteen optimal radiomics features were identified for the multimodal combined model (S1 Fig and S1 Data). The exact 19 selected radiomic features are summarized in S3 Table, including feature names, modalities, filters, classes, and formulas. The variance inflation factor was used to evaluate the multicollinearity among the imaging data features of each model, with values of less than 10 indicating no multicollinearity in these models.

**Fig 3:**
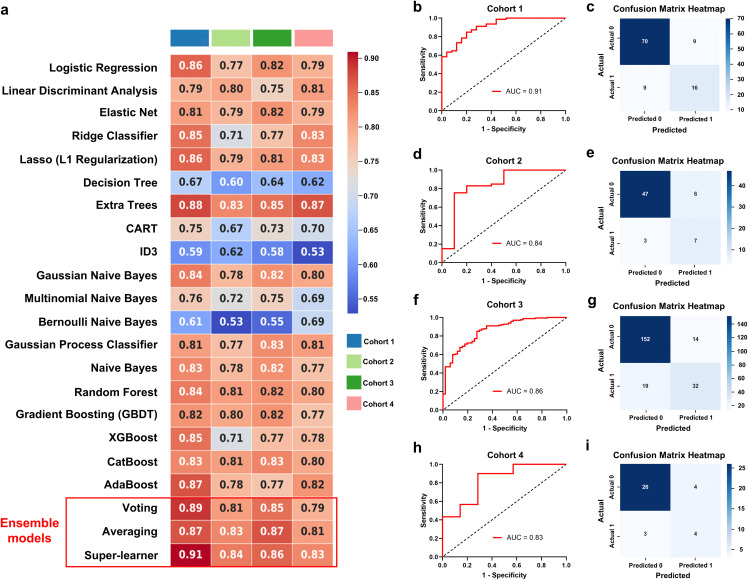
Performance of the radiomics models based on multimodal combined images. (a) 19 individual and 3 ensemble models were constructed based on the combined images (CT, MRI, ^18^F-FDG PET) of cortical foci and evaluated using AUC values across four cohorts. **(b-c)** ROC curves and confusion matrix for SL models in cohort 1. **(d-e)** ROC curves and confusion matrix for SL models in cohort 2. **(f-g)** ROC curves and confusion matrix for SL models in cohort 3. (h-i) ROC curves and confusion matrix for SL models in cohort 4. ROC, receiver operating characteristic curve; AUC, area under the ROC curve.

**Fig 4:**
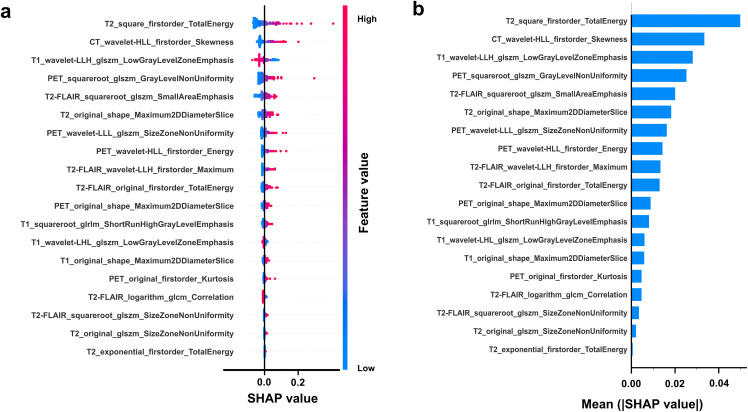
SHAP analysis of the radiomics models based on multimodal combined images. **(a)** Beeswarm plot for selected 19 radiomics features in multimodal combined images of cortical foci. **(b)** Bar plot illustrating how each radiomics feature contributes to prediction. SHAP, shapley additive explanations; glszm, Gray Level Size Zone Matrix; glrlm, Gray Level Run Length Matrix; glcm, Gray Level Co-occurrence Matrix.

Nineteen individual and three ensemble models were developed based on the radiomics features of single-modal images and multimodal combined images. The AUC values demonstrated that these models performed well in the training, validation and test sets, thereby indicating that all of the models were not overfit. Our results revealed that the models based on multimodal combined images (Fig 3) generally performed better than single-modal images of CT (S2 Fig), T1 (S3 Fig), T2 (S4 Fig), T2 FLAIR (S5 Fig), and ^18^F-FDG PET (S6 Fig) in both ML models. The ensemble SL model performed better than the individual models and the ensemble voting and averaging models (Fig 3; S2-S6 Figs; Table 2). To interpret the performance of the SL model, SHAP values were used to represent the contributions of different radiomics features (Fig 4).

**Table 2:** Performance of SL model based on single-modal, combined radiomics and clinical-radiomics.

Features	Group	Performance of model						
		AUC	95%CI	Accuracy	Precision	Specificity	Sensitivity	F1 score
**Radiomics of CT**	Cohort 1	0.78	0.67 - 0.88	0.79	0.55	0.88	0.64	0.59
	Cohort 2	0.71	0.54 - 0.87	0.86	0.55	0.92	0.60	0.57
	Cohort 3	0.71	0.63 - 0.79	0.80	0.57	0.87	0.59	0.58
	Cohort 4	0.73	0.53 - 0.93	0.81	0.50	0.90	0.57	0.53
**Radiomics of T1**	Cohort 1	0.80	0.69 - 0.91	0.79	0.55	0.85	0.64	0.59
	Cohort 2	0.76	0.58 - 0.94	0.87	0.54	0.94	0.70	0.61
	Cohort 3	0.80	0.74 - 0.87	0.91	0.70	0.93	0.71	0.71
	Cohort 4	0.76	0.60 - 0.93	0.84	0.50	0.90	0.57	0.53
**Radiomics of T2**	Cohort 1	0.81	0.72 - 0.91	0.81	0.62	0.89	0.64	0.63
	Cohort 2	0.82	0.66 - 0.98	0.86	0.50	0.92	0.60	0.55
	Cohort 3	0.78	0.71 - 0.85	0.91	0.56	0.96	0.51	0.53
	Cohort 4	0.77	0.60 - 0.94	0.90	0.57	0.90	0.57	0.57
**Radiomics of T2flair**	Cohort 1	0.79	0.67 - 0.90	0.79	0.55	0.84	0.64	0.59
	Cohort 2	0.82	0.67 - 0.97	0.87	0.63	0.92	0.70	0.66
	Cohort 3	0.83	0.76 - 0.90	0.89	0.54	0.86	0.51	0.53
	Cohort 4	0.80	0.63 - 0.97	0.84	0.50	0.89	0.57	0.53
**Radiomics of PET**	Cohort 1	0.86	0.78 - 0.94	0.85	0.59	0.92	0.76	0.67
	Cohort 2	0.81	0.66 - 0.96	0.86	0.38	0.91	0.60	0.47
	Cohort 3	0.85	0.79 - 0.91	0.87	0.65	0.89	0.65	0.65
	Cohort 4	0.79	0.62 - 0.96	0.92	0.67	0.96	0.86	0.75
**Combined Radiomics**	Cohort 1	0.91	0.85 - 0.97	0.84	0.64	0.89	0.64	0.64
	Cohort 2	0.84	0.68 - 1	0.81	0.54	0.89	0.70	0.61
	Cohort 3	0.86	0.80 - 0.92	0.84	0.70	0.92	0.63	0.66
	Cohort 4	0.83	0.65 - 1	0.77	0.50	0.87	0.57	0.53
**Clinical-Radiomics**	Cohort 1	0.92	0.86 - 0.98	0.85	0.76	0.89	0.76	0.76
	Cohort 2	0.92	0.83 - 1	0.89	0.67	0.94	0.60	0.63
	Cohort 3	0.91	0.86 - 0.96	0.90	0.88	0.97	0.69	0.77
	Cohort 4	0.87	0.75 - 0.99	0.86	0.60	0.87	0.86	0.71

SL, super-learner; CT, computed tomography; T1, T1-weighted Imaging; T2, T2-weighted Imaging; T2flair, T2 Fluid-Attenuated Inversion Recovery; PET, positron emission tomography.

### Performance of clinical-radiomics models for predicting epileptogenic foci

**Fig 5:**
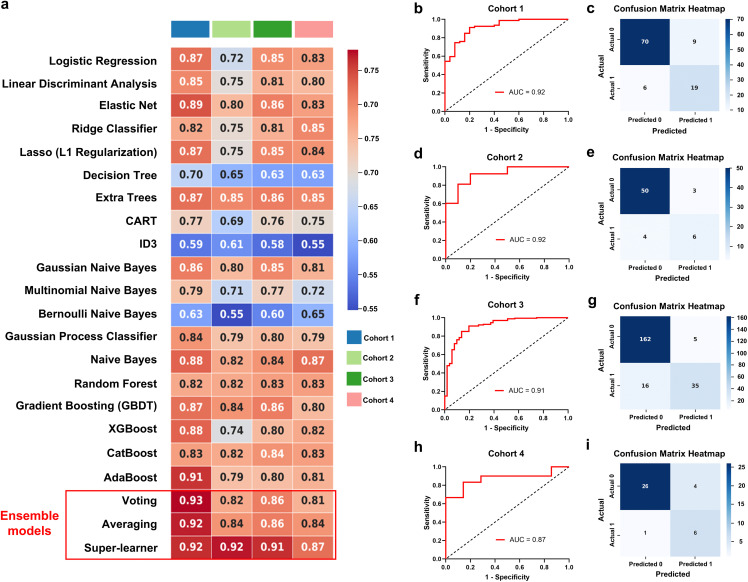
Performance of clinical-radiomics models based on clinical features and multimodal combined images. (a) 19 individual and 3 ensemble models were constructed based on clinical features and multimodal combined images of cortical foci and evaluated using AUC values across four cohorts. **(b-c)** ROC curves and confusion matrix for SL models in cohort 1. **(d-e)** ROC curves and confusion matrix for SL models in cohort 2. **(f-g)** ROC curves and confusion matrix for SL models in cohort 3. (h-i) ROC curves and confusion matrix for SL models in cohort 4. ROC, receiver operating characteristic curve; AUC, area under the ROC curve.

**Fig 6:**
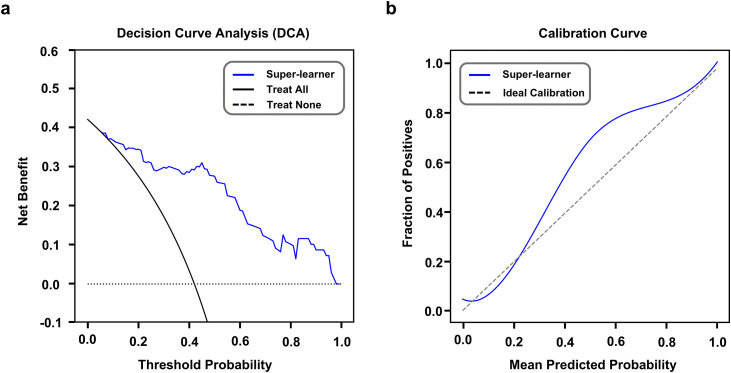
Decision curve and calibration curve of clinical-radiomics SL model. **(a)** Decision curve for the probability of predicting epileptogenic foci using SL model. **(b)** Calibration curve for SL model to identify epileptogenic foci.

Clinical features of cortical foci are essential for the identification of epi foci in the TSC. The volume, SUVmean and TLG were observed to be different between the epi and non-epi foci and could be potential markers for identifying epi foci (Table 1). The predictive ability of the clinical features demonstrated the worse AUC than the model based on the radiomics features in all cohorts (S7 Fig). Furthermore, the three clinical features and nineteen selected multimodal radiomics features were used to construct the multimodal clinical-radiomics models. These multimodal clinical-radiomics models performed well in predicting the epi foci than models based only on clinical or radiomics features (Figs 3 and 5; S7 Fig), and the SL model generally demonstrated the best predictive performance (AUCs: 0.92, 0.92, 0.91 and 0.87 in cohorts 1, 2, 3, and 4, respectively) compared with the individual and ensemble (voting and averaging) models, with p < 0.05 in DeLong’s test. DCA demonstrated that the clinical-radiomics SL model demonstrated good benefits in predicting epi foci when the threshold was greater than 0.05 (Fig 6a). The calibration curve also revealed that the clinical-radiomics SL model achieved good diagnostic performance (Fig 6b). In addition, the clinical-radiomics SL model also demonstrated better performance compared to predictive models that have been previously reported by others (S4 Table, model 1–3) and our team (S4 Table, model 4), a difference that was statistically significant (p < 0.05, DeLong’s test).

### Performance of the clinical-radiomics SL model for predicting surgical outcomes

**Fig 7:**
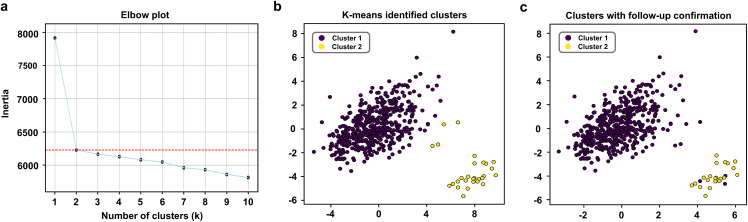
K-means clustering of non-epi foci. **(a)** Elbow plots used to select the number of clusters following k-means clustering. **(b)** K-means-derived clusters of cortical foci. **(c)** K-means-derived clusters with follow-up confirmation.

The multimodal clinical-radiomics SL model exhibited good predictive performance for the 1-year surgical outcomes (AUC = 0.93, Acc = 0.86), 3-year surgical outcomes (AUC = 0.91, Acc = 0.87) and 5-year surgical outcomes (AUC = 0.92, Acc = 0.89) of cortical foci (Table 3). Postoperative seizure recurrence in patients with TSC results from the epileptogenic transformation of non-epi tubers. Furthermore, k-means clustering was used to classify the non-epi tubers during the first preoperative evaluation based on multimodal clinical and radiomics features (Fig 7). Non-epi tubers were divided into two clusters according to elbow plots. Postoperative follow-up data demonstrated that the epi tubers that transformed from non-epi tubers were mainly included in cluster 2 (18/30, S8 Fig), thus indicating that cluster 2 of non-epi foci may exhibit potential epileptogenicity. The clustering results demonstrated that the selected clinical-radiomics features could reflect the true epileptogenicity of cortical foci and further confirmed the ability of the clinical-radiomics SL model to predict long-term surgical outcomes in patients with TSC.

**Table 3:** Performance of SL model in the prediction of long-term outcomes of cortical foci.

Characteristics	Performance of SL model						
	AUC	95%CI	Accuracy	Precision	Specificity	Sensitivity	F1 score
1-year outcomes	0.93	0.84 - 0.97	0.86	0.76	0.90	0.79	0.78
3-year outcomes	0.91	0.81 - 0.94	0.87	0.77	0.92	0.80	0.78
> 5-year outcomes	0.92	0.85 - 0.96	0.89	0.77	0.91	0.82	0.79

SL, super-learner; AUC, area under the curve; 95%CI, 95% confidence interval**.**

### Clinical application of a web-based tool

**Fig 8:**
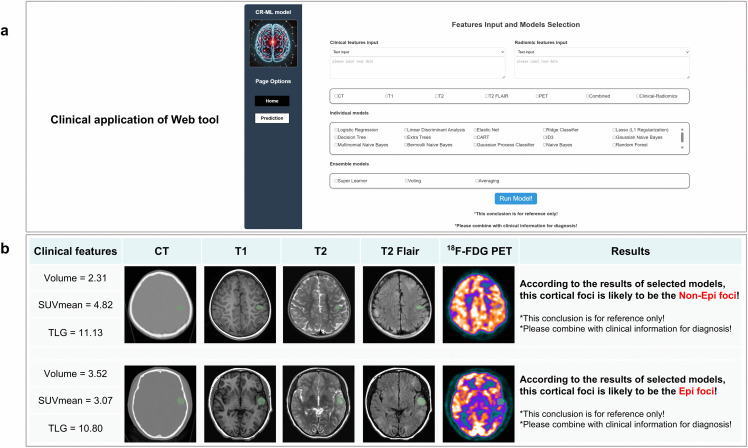
Schematic diagram illustrating an example implementation of the web-based tool. **(a)** Operation interface of the web-based CR-ML model. **(b)** Clinical application example of the CR-ML model for predicting epi and non-epi foci. Clinical and radiomics features are extracted and input into the CR-ML model. Based on clinical requirements, the appropriate models are selected, run, and predictive results are output.

In this study, a web-based tool known as the Clinical-Radiomics-ML (CR-ML) tool (which integrates nineteen individual and three ensemble ML models) was developed. CR-ML can assist clinicians in predicting epi foci during preoperative evaluation. The home page shows a description of the CR-ML, and the prediction page provides the operator interface for clinicians to predict epi foci (Fig 8a, S1 File). First, clinicians are required to input the clinical and radiomics features of cortical foci in Excel or text formats. Second, the target models (including the individual and/or ensemble ML models) are selected. Finally, CR-ML begins to run based on the input data and selected models, after which it generates the prediction results (epi or non-epi foci). Examples of the clinical application of CR-ML are shown in Fig 8b. In addition, 300 cortical foci were randomly selected, and three junior clinicians (5 years of clinical practice) and three senior clinicians (10 years of clinical practice) who were unaware of the epileptogenicity of the cortical foci were invited to make a diagnosis with the aid of CR-ML. The results revealed that the accuracy of the junior clinicians was clearly improved (Acc: from 0.61 to 0.78) with the assistance of CR-ML and even nearly matched the accuracy of the senior clinicians (Acc: 0.80). The accuracy of the senior clinicians also improved (Acc: from 0.80 to 0.87) with the assistance of the CR-ML model (Table 4).

**Table 4:** Performance of CR-ML tool in clinical assistance.

Group	SL model	Junior	Junior with SL model	Senior	Senior with SL model
Accuracy	0.85	0.61	0.78	0.80	0.87
Sensitivity	0.76	0.51	0.72	0.75	0.79
Specificity	0.89	0.64	0.69	0.82	0.89

CR-ML, clinical radiomics-machine learning; Super-learner; Junior Doctors, doctors with 5 years of clinical practice; Senior Doctors, doctors with 10 years of clinical practice.

## Discussion

In this study, compared with individual or other ensemble models, the multimodal clinical-radiomics SL model demonstrated better performance for predicting epi foci. This model represents an accurate and reliable predictive tool that is available for preoperative assessment and neurosurgical management in patients with TSC. Interestingly, this SL model predicted the long-term surgical outcomes of cortical foci. Furthermore, a web-based tool was developed based on the clinical-radiomics models, and its application improved the accuracy of clinicians in the identification of epi foci. This clinical-radiomics SL model promotes the preoperative evaluation of TSC to a noninvasive, precise, and intelligent level; moreover, its clinical application provides an AI-driven method and auxiliary reference for clinicians in the prediction of preoperative epileptogenic foci.

In recent decades, neuroimaging techniques have been used to evaluate brain function and detect abnormalities in various brain disorders. This offers high sensitivity, specificity, and accuracy for detecting foci in neurological diseases. Advances in neuroimaging and the development of data postprocessing methods have resulted in increased accuracy regarding the delineation of epi foci [32]. ^18^F-FDG PET, MRI, and CT are widely used in the presurgical assessment of patients with DRE. PET is particularly essential when MRI and EEG results are discordant or inconclusive, and it can accurately lateralize and classify MRI-negative epi foci [33–35]. Epi foci typically exhibit hypometabolism (lower SUV) on ^18^F-FDG PET during the interictal period of epilepsy, and interictal PET is recommended for the preoperative evaluation of epileptic patients to identify epi zones [36–41]. Previous studies have demonstrated that interhemispheric metabolic asymmetry on interictal PET indicates epi zone locations, and PET-derived indices (such as the SUV, SUV asymmetry features, and oxygen-glucose index) are reliable for identifying potential epi zones [42–46]. Our previous research confirmed that the SUVmean could predict the localization of epi foci in patients with focal cortical dysplasia type IIb and TSC [47]. Both our research and other studies have validated that ^18^F-FDG PET image-based AI models can be used to noninvasively identify epi foci and predict the long-term outcomes of cortical foci in TSC patients [48]. In this study, the predictive models based on ^18^F-FDG PET images performed better than single-modal images of CT, T1, T2, and T2 FLAIR, which further confirmed the ability of ^18^F-FDG PET to consistently distinguish epi foci from non-epi foci.

Multimodal medical imaging combines information from various imaging modalities to provide a comprehensive understanding of clinical diagnosis and research; moreover, it integrates the location of abnormal foci on a map of a patient’s brain. Notably, multimodal image integration is superior for delineating epi zones, enhancing SEEG electrode trajectory planning, and assisting in noninvasive/invasive data interpretation [49]. Furthermore, multimodal ML has been used to predict the incidence of depression in patients with epilepsy and individual postoperative seizure recurrence [50,51]. Previous studies have reported that the automated detection of FCD with high sensitivity and few false-positive findings is feasible based on multimodal data [52]. Moreover, multimodality imaging (including FDG and T2 FLAIR MRI) can improve the detection of epi foci in TSC [53]. Our results also demonstrated that the performance of the ML models based on multimodal images (^18^F-FDG PET, MRI, and CT) was significantly better than that of the single-modal imaging models. These results further confirm the superiority of multimodal images in epi foci prediction.

Radiomics is a novel technique that can extract quantitative features from medical images and detect crucial imaging biomarkers that cannot be visually detectable by clinicians. It provides a basis for establishing brain networks and enhances the quantitative analysis of medical images to support clinical decision-making [54]. Previous research has conceptualized epilepsy as a disruption of the entire brain network, with radiomics demonstrating excellent diagnostic performance in temporal lobe epilepsy using MRI and PET images [55,56]. In this study, radiomics features were extracted from the cortical foci of CT, MRI, and ^18^F-FDG PET images, selected via LASSO regression, and used to develop predictive ML models. The AUC of the model (AUC: 0.86) that was developed by using the radiomics features of ^18^F-FDG PET was significantly greater than that of the previously described model (AUC: 0.77), which was constructed by using quantitative indices [27]. SHAP can be used to explore and identify patterns within complex ML algorithms, along with visualizing the overall or individual contributions of radiomics features and promoting the clinical application of radiomics models [57]. In this study, SHAP-based analysis revealed that radiomics features from PET and T2 FLAIR images were crucial for identifying epi foci. Clinical characteristics of the cortical foci provide direct diagnostic evidence for clinicians; moreover, cortical foci greater than 3–4 cm in size and containing a nidus of calcification are likely to be epi foci [3]. The volume, SUV and TLG of clinical features clearly differed between epi foci and non-epi foci in this study, and integrated clinical-radiomics models exhibited significantly improved performance compared to models that are solely based on radiomics features.

ML can identify complex patterns in high-dimensional data that are not directly evident to human observers; additionally, it can be used to predict future outcomes and offer diagnoses in a wide range of diseases. Based on its capacity for dimensionality reduction and variable selection in large datasets, ML can be used to identify and incorporate the most critical variables in model construction. The critical question in the clinical application of ML is which algorithm is the best, and the choices are significantly influenced by personal preferences and inherent research biases, thus potentially impacting the objectivity of the methodology. In this study, the framework (including 19 common individual ML models and 3 ensemble ML models) was used to construct a predictive model for epi foci. This framework minimizes the bias of model selection and produces greater objectivity. Ensemble ML models combine predictions from multiple individual models into a single model, which is particularly useful for researchers with respect to overcoming difficulties in selecting models with optimal performance [58]. Our results demonstrated that ensemble models performed better than individuals, and the SL model demonstrated the best performance compared to the voting and averaging models. SL is a stacking learning algorithm that exhibits asymptotic performance, as well as the best-weighted combination of the specified base learners [59]. In recent studies, this model has been used to predict chemical acute toxicity in rats [60], type 1 diabetes in children [61], and liver fibrosis in NAFLD [58]; moreover, it has demonstrated the “best-in-class” predictive performance in all individual and ensemble ML models. Epi foci identification constitutes a predictive task, and our studies revealed that the SL model outperforms the individual and other ensemble models in predicting epi foci. The DCA and calibration curves were constructed in this study to confirm the good performance of the SL model. These results further demonstrate the strong interpretability and clinical utility of the SL model in predicting epi foci.

Although deep learning (DL) approaches are widely used in clinical practice and may offer superior performance in many aspects, several considerations led us to choose ML over DL in this study. First, compared with the DL models, the ML models demonstrated superior interpretability. In the clinical application of radiomics features for predicting epi zones, the decision-making process is crucial, as it provides insights into the model’s predictive performance. DL models act as “black boxes”, thus complicating the interpretation of their decision-making processes. Second, the ML models exhibited higher computational efficiencies and versatilities. DL models typically require substantial computational resources and time for training, particularly when handling high-dimensional data that are commonly analyzed in radiomics. However, the predictive models based on ML algorithms were not only effective but also practical and accessible for a broader range of clinical applications. Third, a previous study revealed that a logistic regression model performed better than a DL model (ANN model) for predicting epi foci [27]. Therefore, compared with DL models, ML models may be superior for predicting epi foci based on clinical and radiomics features.

Several studies have explored predictive models of cortical foci in patients with TSC. According to Akira et al., diffusion tensor imaging (DTI) parameters provide moderate diagnostic performance in the detection of TSC-related epi foci. The maximum apparent diffusion coefficient measurements in cortical foci and perifoci areas yielded an AUC of 0.68 ± 0.05 (sensitivity: 81%; specificity: 44%), whereas the maximum radial diffusivity values demonstrated an AUC of 0.63 ± 0.05 (sensitivity: 84%; specificity: 37%) [62]. Recent studies have demonstrated that a predictive model based on radiomics features can identify cortical foci in patients with TSC, with an AUC of 0.81 (95% confidence interval: 0.78–0.84) being reported in the testing set [63]. In addition, our previous study also demonstrated that a logistic regression model based on ^18^F-FDG PET for epi foci in TSC achieved an AUC of 0.77 [27]. Notably, the SL model that was developed in this study demonstrated exceptional predictive performance, with an AUC of 0.92 and an accuracy of 85.0% being achieved.

Additionally, the SL model performed well in predicting long-term surgical outcomes for cortical foci, and its robust predictive ability was consistently validated across both test cohorts. Epi foci can potentially arise from non-epi tubers during disease progression, and postoperative seizure recurrence in TSC results from the epileptogenic transformation of non-epi tubers. K-means clustering revealed two clusters within non-epi foci, with follow-up data identifying cluster 2 as being potentially epileptogenic. These results indicated that postoperative seizure recurrence in TSC patients may originate from the epileptogenic transformation of cluster 2 non-epi foci. The early identification and intervention of cluster 2 non-epi foci is critical for achieving long-term seizure control in patients with TSC. Furthermore, the clinical-radiomics features effectively captured the underlying epileptogenicity of cortical foci, thereby reinforcing the ability of the SL model to predict surgical outcomes.

To enhance the accessibility and practical application of the predictive model, we developed a web-based tool (CR-ML) by featuring both individual and ensemble models based on clinical-radiomics features. The significance of CR-ML is based on its ability to reduce error from clinical experience and provide more objective and precise guidance during preoperative evaluation. AI tools have been reported to provide assistance for clinical diagnosis. For example, Yi et al. reported that a LiLNet model for the diagnosis of focal liver lesions (including those of hepatocellular carcinoma and intrahepatic cholangiocarcinoma) can aid in clinical diagnosis in regions with a shortage of radiologists [64]. Shan et al. reported that a Claude 2.1 model significantly outperformed residents, fellows, and attendings in addressing colorectal cancer queries [65]. Our results demonstrated that compared with senior clinicians, CR-ML based on the SL model performed better in predicting epi foci; moreover, it aided junior clinicians in improving the predictive accuracy to a level comparable to that of senior clinicians. These results further confirmed the clinical application value of this predictive model.

Furthermore, we compared our web tool with prior online platforms for epilepsy, including EPINOV [66] and SeizureTracker [67]. EPINOV is a clinical research tool that integrates virtual stereotactic EEG simulations into the presurgical workflow for pharmacoresistant focal epilepsies. It leverages individual brain connectomics and biophysical modeling to simulate epileptogenic network dynamics for surgical guidance. In contrast, our tool is specifically trained for TSC patients and employs machine learning frameworks to directly identify epi foci from multiple cortical foci by learning from clinical and radiomics features rather than simulating network dynamics. This approach enables the functions of our model in the prediction of epi foci in TSC patients. SeizureTracker is a patient-facing management platform designed for the comprehensive daily management of epilepsy, enabling users to track seizure events, monitor medication adherence, and generate consolidated health reports. Our tool is designed as a clinician-facing decision-support tool that focuses on identifying epi foci and predicting surgical outcomes. Importantly, our tool is complementary to SeizureTracker. Longitudinal postoperative seizure freedom data recorded on platforms such as SeizureTracker can serve as invaluable data for validating our tool’s predictive accuracy. The integration of multiple AI applications will pave the way for more precise and personalized epilepsy treatment in the future.

We acknowledge several limitations of the present study. First, all of the TSC patients included in this study were diagnosed with intractable epilepsy. This selection criterion introduces spectrum bias, as it systematically excludes patients with less severe, drug-responsive epilepsy, as well as those who have not developed epilepsy at all. Therefore, the predictive features identified with our model are most relevant and representative of the subpopulation with intractable epilepsy. Our model may assist in evaluating epi foci in the broader TSC population, but the inherent selection bias is likely to affect its predictive accuracy. Second, although we included multicenter data from patients with TSC, the sample size was relatively small due to the low incidence of TSC [26]. The annual surgical volume of the 3 centers in this study was 13.1 (4.36 per center), which is reasonable relative to the national estimate (2.23 per center) [3] and exceeds that reported in other international multicenter studies [68–71]. Furthermore, the annual surgical volume of individual centers in this study ranged from 2.2 to 8.3, which are comparable to those in previous single-center studies (ranging from 1.5 to 7.4) [72–76]. Although the sample size is acceptable, more cohorts from comprehensive epilepsy centers, including international cohorts, need to be enrolled to validate and refine our findings. Third, surgical outcomes were assessed by using the ILAE classification and dichotomized into SF or non-SF groups. The classification of these categories into a binary surgical outcome may result in the loss of clinically relevant information, such as the difference between patients with only auras and those with no worthwhile improvement. This may affect the interpretability and generalizability of the model in clinical practice. Future studies could be conducted to develop models that predict outcomes across the full spectrum of ILAE classifications. Fourth, compared with the DL algorithm, the traditional ML algorithm performed better in terms of interpretability, computational efficiency, and generalizability. However, DL models benefit from larger datasets to achieve optimal performance and avoid overfitting, thus making them powerful for dealing with high-dimensional radiomics data [77]. It would be beneficial to develop and validate DL models for predicting epi foci by using larger patient cohorts in future studies.

In conclusion, the clinical-radiomics SL model developed in this study represents a promising tool for guiding preoperative evaluation, predicting surgical outcomes, and facilitating surgical approach decisions. This model effectively addresses diagnostic discrepancies among clinicians of varying expertise levels and enables personalized treatment strategies while minimizing the invasiveness and financial strain of patients. Although our results indicate superior performance compared with existing methods for preoperative evaluation and neurosurgical intervention in patients with TSC, further improvements and investigations enrolling more cohorts from comprehensive epilepsy centers are needed. The clinical-radiomics SL model demonstrates considerable translational potential for the prediction of epi foci in both TSC patients and all epilepsy patients with multiple epi foci.

## Supporting information

S1 Appendix STARD Checklist. (PDF)

S2 Appendix STARD Flow diagram. (TIF)

S3 Appendix PyRadiomics parameter. (YAML)

S1 Data Dataset. Dataset underlying the findings.
(CSV)

S1 Fig Radiomics features selection using the least absolute shrinkage and selection operator (LASSO) regression in single- and multi-modal imaging data. (a-b) Selection of the tuning parameter lambda (λ) via 10-fold cross validation and weight of three resulting features with nonzero coefficients in single CT imaging data. (c-d) Selection of the tuning parameter lambda (λ) via 10-fold cross validation and weight of six resulting features with nonzero coefficients in single T1 imaging data. (e-f) Selection of the tuning parameter lambda (λ) via 10-fold cross validation and weight of five resulting features with nonzero coefficients in single T2 imaging data. (g-h) Selection of the tuning parameter lambda (λ) via 10-fold cross validation and weight of four resulting features with nonzero coefficients in single T2 FLAIR imaging data. (i-j) Selection of the tuning parameter lambda (λ) via 10-fold cross validation and weight of seven resulting features with nonzero coefficients in single ^18^F-FDG PET imaging data. (k-l) Selection of the tuning parameter lambda (λ) via 10-fold cross validation and weight of nineteen resulting features with nonzero coefficients in multimodal combined imaging data. LASSO, least absolute shrinkage and selection operator.
(DOCX)

S2 Fig Performance of the radiomics models based on CT images. (a) 19 individual and 3 ensemble models were constructed based on CT images of cortical foci and evaluated using AUC values across four cohorts. (b-c) ROC curves and confusion matrix for SL models in cohort 1. (d-e) ROC curves and confusion matrix for SL models in cohort 2. (f-g) ROC curves and confusion matrix for SL models in cohort 3. (h-i) ROC curves and confusion matrix for SL models in cohort 4. ROC, receiver operating characteristic curve; AUC, area under the ROC curve.
(DOCX)

S3 Fig Performance of the radiomics models based on T1 images. (a) 19 individual and 3 ensemble models were constructed based on T1 images of cortical foci and evaluated using AUC values across four cohorts. (b-c) ROC curves and confusion matrix for SL models in cohort 1. (d-e) ROC curves and confusion matrix for SL models in cohort 2. (f-g) ROC curves and confusion matrix for SL models in cohort 3. (h-i) ROC curves and confusion matrix for SL models in cohort 4. ROC, receiver operating characteristic curve; AUC, area under the ROC curve.
(DOCX)

S4 Fig Performance of the radiomics models based on T2 images. (a) 19 individual and 3 ensemble models were constructed based on T2 images of cortical foci and evaluated using AUC values across four cohorts. (b-c) ROC curves and confusion matrix for SL models in cohort 1. (d-e) ROC curves and confusion matrix for SL models in cohort 2. (f-g) ROC curves and confusion matrix for SL models in cohort 3. (h-i) ROC curves and confusion matrix for SL models in cohort 4. ROC, receiver operating characteristic curve; AUC, area under the ROC curve.
(DOCX)

S5 Fig Performance of the radiomics models based on T2 FLAIR images. (a) 19 individual and 3 ensemble models were constructed based on T2 FLAIR images of cortical foci and evaluated using AUC values across four cohorts. (b-c) ROC curves and confusion matrix for SL models in cohort 1. (d-e) ROC curves and confusion matrix for SL models in cohort 2. (f-g) ROC curves and confusion matrix for SL models in cohort 3. (h-i) ROC curves and confusion matrix for SL models in cohort 4. ROC, receiver operating characteristic curve; AUC, area under the ROC curve.
(DOCX)

S6 Fig Performance of the radiomics models based on 18F-FDG PET images. (a) 19 individual and 3 ensemble models were constructed based on ^18^F-FDG PET images of cortical foci and evaluated using AUC values across four cohorts. (b-c) ROC curves and confusion matrix for SL models in cohort 1. (d-e) ROC curves and confusion matrix for SL models in cohort 2. (f-g) ROC curves and confusion matrix for SL models in cohort 3. (h-i) ROC curves and confusion matrix for SL models in cohort 4. ROC, receiver operating characteristic curve; AUC, area under the ROC curve.
(DOCX)

S7 Fig Predictive performance of clinical features. (a) Predictive performance of Volume, SUVmean and TLG in cohort 1. (b) Predictive performance of Volume, SUVmean and TLG in cohort 2. (c) Predictive performance of Volume, SUVmean and TLG in cohort 3. (d) Predictive performance of Volume, SUVmean and TLG in cohort 4.
(DOCX)

S8 Fig Cluster agreement matrix evaluating k-means clustering stability. (DOCX)

S1 Table Clinical characteristics of patients with TSC in four cohorts. (DOCX)

S2 Table Scanner models and reconstruction parameters from three different centers. (DOCX)

S3 Table Feature names, modalities, filters, classes, and formulas of the 19 radiomic features. (DOCX)

S4 Table Performance comparison of the SL model with previously reported models. (DOCX)

S1 File Video. Clinical application of the web-tool.
(MP4)

S1 Text Protocol. Study Protocol.
(DOCX)
