# Supplementary material for: Development and validation of interpretable multimodal clinical-radiomics models for predicting epileptogenic foci and surgical outcomes in tuberous sclerosis complex: A multicenter study
Source: PLOS Digit Health. 2026 Feb 26;5(2):e0001259. doi: 10.1371/journal.pdig.0001259 (PMC12944716; doi:10.1371/journal.pdig.0001259)
Supplement: S1 Fig — (a-b) Selection of the tuning parameter lambda (λ) via 10-fold cross validation and weight of three resulting features with nonzero coefficients in single CT imaging data. (c-d) Selection of the tuning parameter lambda (λ) via 10-fold cross validation and weight of six resulting features with nonzero coefficients in single T1 imaging data. (e-f) Selection of the tuning parameter lambda (λ) via 10-fold cross validation and weight of five resulting features with nonzero coefficients in single T2 imaging data. (g-h) Selection of the tuning parameter lambda (λ) via 10-fold cross validation and weight of four resulting features with nonzero coefficients in single T2 FLAIR imaging data. (i-j) Selection of the tuning parameter lambda (λ) via 10-fold cross validation and weight of seven resulting features with nonzero coefficients in single 18F-FDG PET imaging data. (k-l) Selection of the tuning parameter lambda (λ) via 10-fold cross validation and weight of nineteen resulting features with nonzero coefficients in multimodal combined imaging data. LASSO, least absolute shrinkage and selection operator. (DOCX) [file pdig.0001259.s005.docx]

**

S1 Fig. Radiomics features selection using the LASSO regression in single- and multi-modal imaging data.** (a-b) Selection of the tuning parameter lambda (λ) via 10-fold cross validation and weight of three resulting features with nonzero coefficients in single CT imaging data. (c-d) Selection of the tuning parameter lambda (λ) via 10-fold cross validation and weight of six resulting features with nonzero coefficients in single T1 imaging data. (e-f) Selection of the tuning parameter lambda (λ) via 10-fold cross validation and weight of five resulting features with nonzero coefficients in single T2 imaging data. (g-h) Selection of the tuning parameter lambda (λ) via 10-fold cross validation and weight of four resulting features with nonzero coefficients in single T2 FLAIR imaging data. (i-j) Selection of the tuning parameter lambda (λ) via 10-fold cross validation and weight of seven resulting features with nonzero coefficients in single ^18^F-FDG PET imaging data. (k-l) Selection of the tuning parameter lambda (λ) via 10-fold cross validation and weight of nineteen resulting features with nonzero coefficients in multimodal combined imaging data. LASSO, least absolute shrinkage and selection operator.
