# Supplementary material for: Development and validation of interpretable multimodal clinical-radiomics models for predicting epileptogenic foci and surgical outcomes in tuberous sclerosis complex: A multicenter study
Source: PLOS Digit Health. 2026 Feb 26;5(2):e0001259. doi: 10.1371/journal.pdig.0001259 (PMC12944716; doi:10.1371/journal.pdig.0001259)
Supplement: S3 Fig — (a) 19 individual and 3 ensemble models were constructed based on T1 images of cortical foci and evaluated using AUC values across four cohorts. (b-c) ROC curves and confusion matrix for SL models in cohort 1. (d-e) ROC curves and confusion matrix for SL models in cohort 2. (f-g) ROC curves and confusion matrix for SL models in cohort 3. (h-i) ROC curves and confusion matrix for SL models in cohort 4. ROC, receiver operating characteristic curve; AUC, area under the ROC curve. (DOCX) [file pdig.0001259.s007.docx]

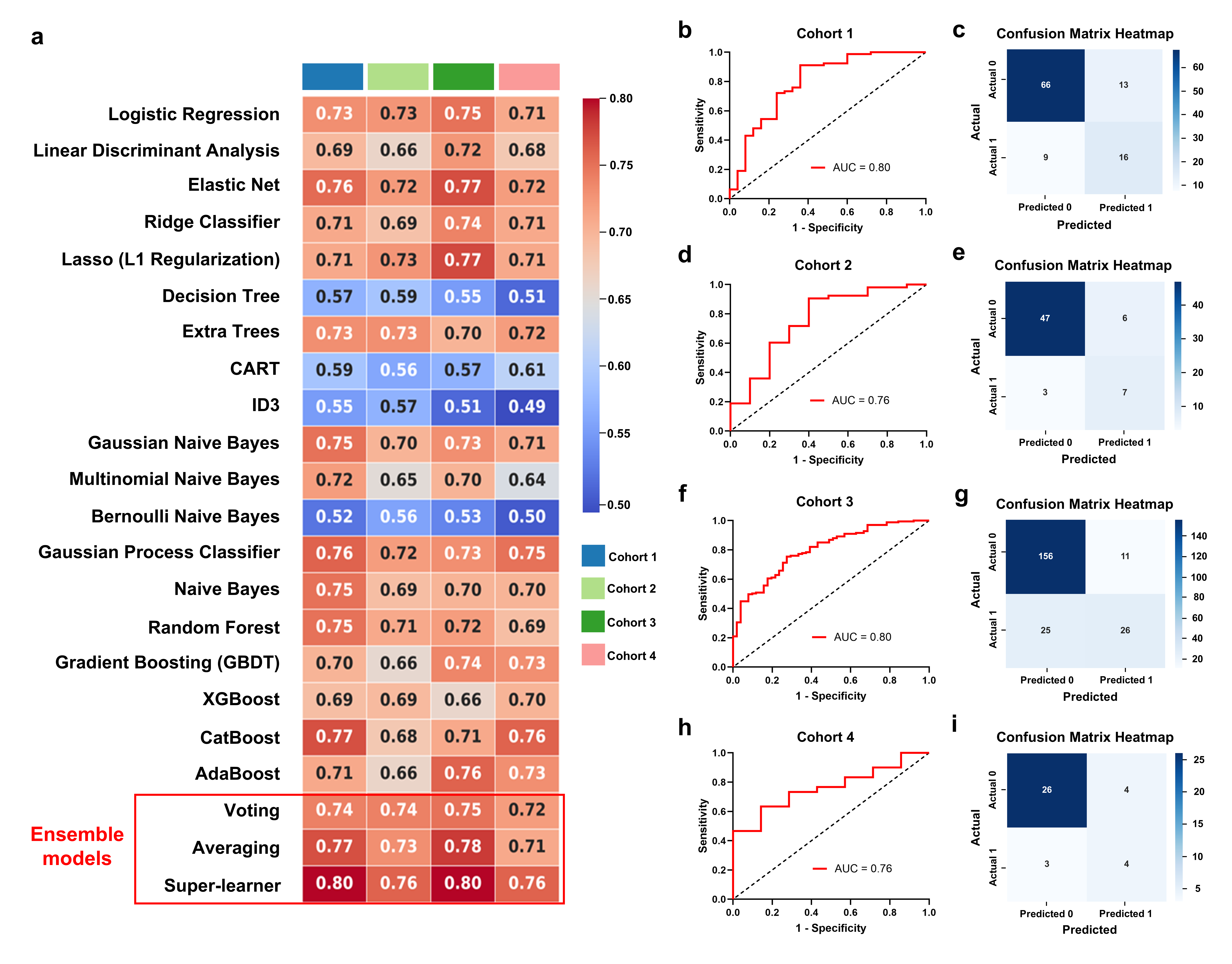


**S3 Fig. Performance of the radiomics models based on T1 images.** (a) 19 individual and 3 ensemble models were constructed based on T1 images of cortical foci and evaluated using AUC values across four cohorts. (b-c) ROC curves and confusion matrix for SL models in cohort 1. (d-e) ROC curves and confusion matrix for SL models in cohort 2. (f-g) ROC curves and confusion matrix for SL models in cohort 3. (h-i) ROC curves and confusion matrix for SL models in cohort 4. ROC, receiver operating characteristic curve; AUC, area under the ROC curve.
