# Supplementary material for: Development and validation of interpretable multimodal clinical-radiomics models for predicting epileptogenic foci and surgical outcomes in tuberous sclerosis complex: A multicenter study
Source: PLOS Digit Health. 2026 Feb 26;5(2):e0001259. doi: 10.1371/journal.pdig.0001259 (PMC12944716; doi:10.1371/journal.pdig.0001259)
Supplement: S7 Fig — (a) Predictive performance of Volume, SUVmean and TLG in cohort 1. (b) Predictive performance of Volume, SUVmean and TLG in cohort 2. (c) Predictive performance of Volume, SUVmean and TLG in cohort 3. (d) Predictive performance of Volume, SUVmean and TLG in cohort 4. (DOCX) [file pdig.0001259.s011.docx]

**
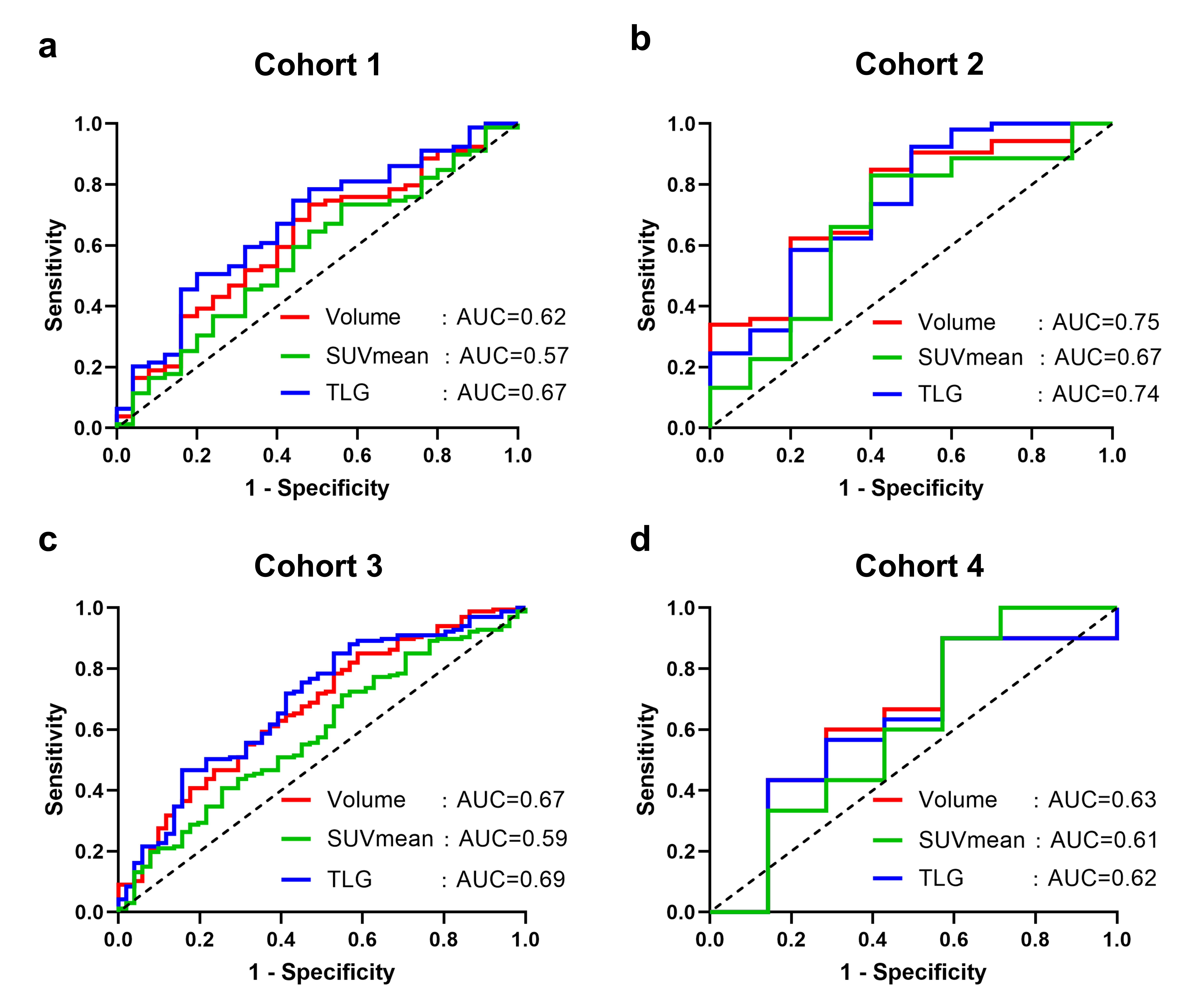
S7** **Fig. Predictive performance of clinical features.** (a) Predictive performance of Volume, SUVmean and TLG in cohort 1. (b) Predictive performance of Volume, SUVmean and TLG in cohort 2. (c) Predictive performance of Volume, SUVmean and TLG in cohort 3. (d) Predictive performance of Volume, SUVmean and TLG in cohort 4.
