# Supplementary figures and images for: Development and validation of interpretable multimodal clinical-radiomics models for predicting epileptogenic foci and surgical outcomes in tuberous sclerosis complex: A multicenter study
Source: PLOS Digit Health. 2026 Feb 26;5(2):e0001259. doi: 10.1371/journal.pdig.0001259 (PMC12944716; doi:10.1371/journal.pdig.0001259)

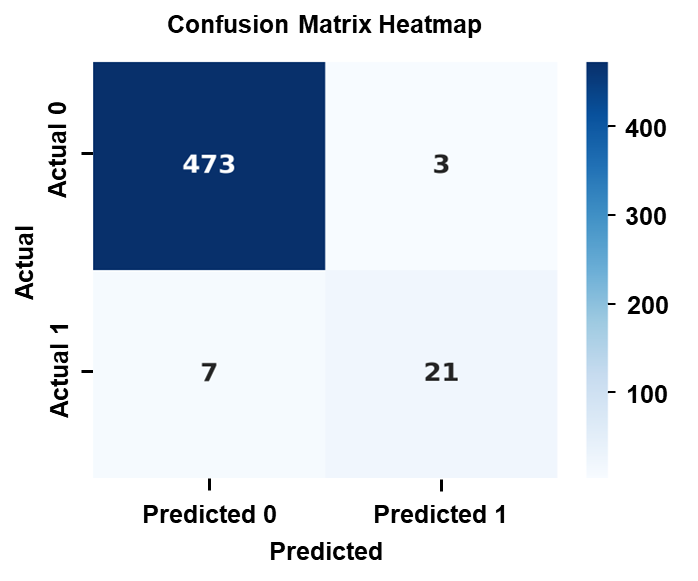
**S8 Fig. Cluster agreement matrix evaluating k-means clustering stability.**

Supplement: S8 Fig — (DOCX) [file pdig.0001259.s012.docx]
