# Supplementary material for: Development and validation of interpretable multimodal clinical-radiomics models for predicting epileptogenic foci and surgical outcomes in tuberous sclerosis complex: A multicenter study
Source: PLOS Digit Health. 2026 Feb 26;5(2):e0001259. doi: 10.1371/journal.pdig.0001259 (PMC12944716; doi:10.1371/journal.pdig.0001259)
Supplement: S1 Table — (DOCX) [file pdig.0001259.s013.docx]

| **S1 Table. Clinical characteristics of patients with TSC in four cohorts.** | | | | | | | | |
| --- | --- | --- | --- | --- | --- | --- | --- | --- |
| **Clinical Characteristics** | | **Cohort 1** |  | **Cohort 2** |  | **Cohort 3** |  | **Cohort 4** |
|  |  | Retrospective cohort of Center 1 (n = 57) |  | Prospective cohort of Center 1 (n = 8) |  | Retrospective cohort of Center 2 (n = 35) |  | Retrospective cohort of Center 3 (n = 6) |
| **Age** | | 12 years (0.5-37) |  | 10 years (0.2-23) |  | 13 years (1-26) |  | 11 years (2-29) |
| **Gender** | |  |  |  |  |  |  |  |
| Female | | 26(45.6%) |  | 4(50.0%) |  | 18(51.4%) |  | 2(33.3%) |
| Male | | 31(54.4%) |  | 4(50.0%) |  | 17(48.6%) |  | 4(66.7%) |
| **Type of variation** | |  |  |  |  |  |  |  |
| TSC1 | | 13(22.8%) |  | 3(37.5%) |  | 8(22.9%) |  | 3(50.0%) |
| TSC2 | | 35(61.4%) |  | 5(62.5%) |  | 14(40.0%) |  | 2(33.3%) |
| No variation | | 9(15.8%) |  | — |  | 13(37.1%) |  | 1(16.7%) |
| **Seizure onset (month)** | | 19.21(1-163) |  | 24.21(7-110) |  | 32.24(1-132) |  | 23.00(16-108) |
| **Seizure frequency** | |  |  |  |  |  |  |  |
| Daily | | 30(52.6%) |  | 4(50.0%) |  | 19(54.3%) |  | 4(66.7%) |
| Weekly | | 13(22.8%) |  | 2(25.0%) |  | 11(31.4%) |  | 2(33.3%) |
| At least twice per month | | 11(19.3%) |  | 2(25.0%) |  | 5(14.3%) |  | — |
| Yearly | | 3(5.3%) |  | — |  | — |  | — |
| **Number of ASMs** | | 2.29(1-4) |  | 3.17(2-5) |  | 2.97(1-6) |  | 2.5(1-4) |
| **Number of Cortical foci** | |  |  |  |  |  |  |  |
| 1-3 | | 11(19.3%) |  | 1(12.5%) |  | 5(14.3%) |  | 1(16.7%) |
| 4-6 | | 34(59.6%) |  | 5(62.5%) |  | 20(57.1%) |  | 3(50.0%) |
| >6 | | 12(21.1%) |  | 2(25.0%) |  | 10(28.6%) |  | 2(33.3%) |
| **Ictal epileptic discharges** | |  |  |  |  |  |  |  |
| Focal | | 13(22.8%) |  | 1(12.5%) |  | 5(14.3%) |  | 1(16.7%) |
| Lateralized | | 16(28.1%) |  | 2(25.0%) |  | 13(37.1%) |  | 2(33.3%) |
| Multi-focal | | 18(31.6%) |  | 3(37.5%) |  | 5(14.3%) |  | 1(16.7%) |
| Generalized | | 10(17.5%) |  | 2(25.0%) |  | 12(34.3%) |  | 2(33.3%) |
| **Surgery approach** | |  |  |  |  |  |  |  |
| (Multiple) tuberectomy | | 23(40.4%) |  | 4(50.0%) |  | 18(51.4%) |  | 3(50.0%) |
| (Multiple) lobectomy | | 15(26.3%) |  | 1(12.5%) |  | 8(22.9%) |  | 1(16.7%) |
| Lobectomy + tuberectomy | | 19(33.3%) |  | 3(37.5%) |  | 9(25.7%) |  | 2(33.3)% |
| **Surgical outcomes** | |  |  |  |  |  |  |  |
| 1 year follow | SF | 45(78.9%) |  | 7(87.5%) |  | 30(85.7%) |  | 5(83.3%) |
|  | No-SF | 12(21.1%) |  | 1(12.5%) |  | 5(14.3%) |  | 1(16.7%) |
| 3 years follow | SF | 36(78.3%) |  | **—** |  | 28(82.4%) |  | 1(50%) |
|  | No-SF | 10(21.7%) |  | **—** |  | 6(17.6%) |  | 1(50%) |
| >5 years follow | SF | 17(65.4%) |  | **—** |  | 9(69.2%) |  | **—** |
|  | No-SF | 9(34.6%) |  | **—** |  | 4(30.8%) |  | **—** |
| TSC, tuberous sclerosis complex; SUV, standard uptake value; ASM, anti-seizure medication; SF, seizure freedom; No-SF, No-seizure freedom. | | | | | | | | |
