# Supplementary material for: Development and validation of interpretable multimodal clinical-radiomics models for predicting epileptogenic foci and surgical outcomes in tuberous sclerosis complex: A multicenter study
Source: PLOS Digit Health. 2026 Feb 26;5(2):e0001259. doi: 10.1371/journal.pdig.0001259 (PMC12944716; doi:10.1371/journal.pdig.0001259)
Supplement: S2 Table — (DOCX) [file pdig.0001259.s014.docx]

| **S2 Table Scanner modalities, vendors and parameters from three different centers.** | | | | |
| --- | --- | --- | --- | --- |
| **Center** | **Modality** | **Vendor** | **Parameters** |  |
| Center 1  (Chongqing, China) | CT | Philips Medical Systems | Model: GEMINI Astonsh TF-64  Reconstruction algorithm: iDose⁴  Slice thickness: 0.625 mm |  |
|  | MRI | Philips Medical Systems | Model: Ingenia  Magnetic field strength: 3.0 T  Coil type: Head/Body phased-array coil  Reconstruction algorithm: Compressed SENSE |  |
|  | PET | Philips Medical Systems | Model: GEMINI Astonsh TF-64  Reconstruction algorithm: TOF-OSEM  Spatial resolution: 4.2 mm |  |
| Center 2  (Guangdong, China) | CT | GE Medical Systems | Model: Discovery ST  Reconstruction algorithm: ASiR  Slice thickness: 0.625 mm |  |
|  | MRI | GE Medical Systems | Model: SIGNA Pioneer  Magnetic field strength: 3.0 T  Coil type: Head/body phased-array coil;  Reconstruction algorithm: AIR Recon DL |  |
|  | PET | GE Medical Systems | Model: Discovery ST  Reconstruction algorithm: VUE Point FX  Spatial resolution: 4.0 mm |  |
| Center 3  (Chengdu, China) | CT | SIEMENS | Model: BIOGRAPH 64  Reconstruction algorithm: Filtered Back-Projection;  Slice thickness: 0.625 mm |  |
|  | MRI | GE Medical Systems | Model: SIGNA Pioneer  Magnetic field strength: 3.0 T  Coil type: Head/body phased-array coil  Reconstruction algorithm: AIR Recon DL |  |
|  | PET | SIEMENS | Model: BIOGRAPH 64  Reconstruction algorithm: OSEM2D 4i24s  Spatial resolution: 4.2 mm |  |
| CT, computed tomography; MRI, magnetic resonance imaging; PET, positron emission tomography. | | | |  |
