# Supplementary material for: Development and validation of interpretable multimodal clinical-radiomics models for predicting epileptogenic foci and surgical outcomes in tuberous sclerosis complex: A multicenter study
Source: PLOS Digit Health. 2026 Feb 26;5(2):e0001259. doi: 10.1371/journal.pdig.0001259 (PMC12944716; doi:10.1371/journal.pdig.0001259)
Supplement: S3 Table — (DOCX) [file pdig.0001259.s015.docx]

| **S3 Table. Feature names, modalities, filters, classes, and formulas of the 19 radiomic features.** | | | | |
| --- | --- | --- | --- | --- |
| **Feature Name** | **Modality** | **Filter** | **Class** | **Formula** |
| TotalEnergy | T2 | square | firstorder | $\sum_{i=1}^{N} I_{i}^{2}$ |
| Skewness | CT | wavelet-HLL | firstorder | $\frac{\sum_{i=1}^{N} \left( I_{i}-\mu\right)^{3}}{N\sigma^{3}}$ |
| GrayLevelNonUniformity | PET | squareroot | glszm | $\frac{\sum_{j=1}^{N_{g}} \left( \sum_{i=1}^{N_{z}} P(i,j) \right)^{2}}{N_{z}}$ |
| LowGrayLevelZoneEmphasis | T1 | wavelet-LLH | glszm | $\frac{\sum_{i,j} \frac{P(i,j)}{j^{2}}}{N_{z}}$ |
| SmallAreaEmphasis | T2 FLAIR | squareroot | glszm | $\frac{\sum_{i,j} \frac{P(i,j)}{i^{2}}}{N_{z}}$ |
| SizeZoneNonUniformity | PET | wavelet-LLL | glszm | $\frac{\sum_{i=1}^{N_{z}} \left( \sum_{j=1}^{N_{g}} P(i,j) \right)^{2}}{N_{z}}$ |
| Maximum2DDiameterSlice | T2 | original | shape | $\max_{i\in\text{slices}}\left( \max_{p,q\in S_{i}}d(p,q) \right)$ |
| Energy | PET | wavelet-HLL | firstorder | $\sum_{i=1}^{N} I_{i}^{2}$ |
| Maximum | T2 FLAIR | wavelet-LLH | firstorder | $max(I_{i})$ |
| TotalEnergy | T2 FLAIR | original | firstorder | $\sum_{i=1}^{N} I_{i}^{2}$ |
| Maximum2DDiameterSlice | PET | original | shape | $\max_{i\in\text{slices}}\left( \max_{p,q\in S_{i}}d(p,q) \right)$ |
| ShortRunHighGrayLevelEmphasis | T1 | squareroot | glrlm | $\frac{\sum_{i,j} \frac{j^{2}P(i,j)}{i^{2}}}{N_{r}}$ |
| Kurtosis | PET | original | firstorder | $\frac{\frac{1}{N}\sum(I_{i}-\mu)^{4}}{\sigma^{4}}$ |
| Maximum2DDiameterSlice | T1 | original | shape | $\max_{i\in\text{slices}}\left( \max_{p,q\in S_{i}}d(p,q) \right)$ |
| LowGrayLevelZoneEmphasis | T1 | wavelet-LHL | glszm | $\frac{\sum_{i,j} \frac{P(i,j)}{j^{2}}}{N_{z}}$ |
| Correlation | T2 FLAIR | logarithm | glcm | $\frac{\sum_{i,j} (i-\mu_{i})(j-\mu_{j})P(i,j)}{\sigma_{i}\sigma_{j}}$ |
| SizeZoneNonUniformity | T2 FLAIR | squareroot | glszm | $\frac{\sum_{i=1}^{N_{z}} \left( \sum_{j=1}^{N_{g}} P(i,j) \right)^{2}}{N_{z}}$ |
| SizeZoneNonUniformity | T2 | original | glszm | $\frac{\sum_{i=1}^{N_{z}} \left( \sum_{j=1}^{N_{g}} P(i,j) \right)^{2}}{N_{z}}$ |
| TotalEnergy | T2 | exponential | firstorder | $\sum_{i=1}^{N} I_{i}^{2}$ |
| CT, computed tomography; T1, T1-weighted Imaging; T2, T2-weighted Imaging; T2 FLAIR, T2 Fluid-Attenuated Inversion Recovery; PET, positron emission tomography. | | | | |
