# Supplementary material for: Development and validation of interpretable multimodal clinical-radiomics models for predicting epileptogenic foci and surgical outcomes in tuberous sclerosis complex: A multicenter study
Source: PLOS Digit Health. 2026 Feb 26;5(2):e0001259. doi: 10.1371/journal.pdig.0001259 (PMC12944716; doi:10.1371/journal.pdig.0001259)
Supplement: S4 Table — (DOCX) [file pdig.0001259.s016.docx]

| **S4 Table. Performance comparison of the SL model with previously reported models.** | | | | | | | |
| --- | --- | --- | --- | --- | --- | --- | --- |
| **Features** | **Performance of SL model** | | | | | | |
|  | AUC | 95%CI | Accuracy (%) | Precision (%) | Specificity (%) | Sensitivity (%) | F1 score (%) |
| Model 1 | 0.68 | 0.63 - 0.73 | — | — | 0.44 | 0.81 | — |
| Model 2 | 0.63 | 0.58 - 0.68 | — | — | 0.37 | 0.84 | — |
| Model 3 | 0.81 | 0.78 - 0.84 | — | — | 0.91 | 0.60 | — |
| Model 4 | 0.77 | 0.70 - 0.83 | 0.78 | — | — | 0.84 | — |
| SL model | 0.92 | 0.86 - 0.98 | 0.85 | 0.76 | 0.89 | 0.76 | 0.76 |
| SL, super-learner; AUC, area under the curve; 95%CI, 95% confidence interval. | | | | | | | |
