# Supplementary material for: Development and validation of interpretable multimodal clinical-radiomics models for predicting epileptogenic foci and surgical outcomes in tuberous sclerosis complex: A multicenter study
Source: PLOS Digit Health. 2026 Feb 26;5(2):e0001259. doi: 10.1371/journal.pdig.0001259 (PMC12944716; doi:10.1371/journal.pdig.0001259)
Supplement: S1 Text — Study Protocol. (DOCX) [file pdig.0001259.s018.docx]

| **Study Protocol** | | |
| --- | --- | --- |
| 1. **Title** | Development and validation of interpretable multimodal clinical-radiomics models for predicting epileptogenic foci and surgical outcomes in tuberous sclerosis complex: a multicenter study | |
| 1. **Person responsible** | Zhongke Wang  Army Medical University | Gender of the person responsible:  Female  Male  Divers |
| 1. **Institution** | Comprehensive Epilepsy Center, Department of Neurosurgery, Xinqiao Hospital, Army Medical University | |
| 1. **Background** | Tuberous sclerosis complex (TSC) is a multisystem, autosomal dominant syndrome affecting approximately 1 in 6,000 live births. Epilepsy, which occurs in 80–90% of patients with TSC, is the most common neurological manifestation of this disease, with nearly two-thirds of these patients developing drug-resistant epilepsy (DRE) (1, 2). As reported in the 2021 updated international diagnostic criteria for TSC (2), “multiple cortical tubers” in an individual patient are a characteristic feature of TSC, and it has been used to replace cortical dysplasia in diagnostic criteria, thus suggesting the pivotal role of multiple cortical foci in the epileptogenesis of TSC. Previous studies have demonstrated that nearly all TSC patients exhibit multiple cortical tubers, which serve as potential seizure-onset zones, and resection of epi foci is the most effective intervention for intractable epilepsy in TSC patients (3). The precise preoperative localization of epi foci among multiple cortical tubers is crucial for determining resection strategies, surgical outcomes, and prognosis in TSC-related epilepsy, yet remains a significant clinical challenge (4). Although intracranial electroencephalography (EEG) remains the gold standard for the identification of epi foci, its invasive nature and expense limit its clinical application. Noninvasive neuroimaging methods, including computed tomography (CT), magnetic resonance imaging (MRI), and ^18^F-fluorodeoxyglucose positron emission tomography (^18^F-FDG PET), are increasingly being used as imaging biomarkers to predict epi foci. The combination of multimodal features of MRI with PET has proven to be valuable in detecting temporal lobe epilepsy with dual pathology (5). Advances in neuroimaging are crucial for identifying cortical malformations that cause seizure disorders. Neuroimaging gradient alterations in FCD II can be used to guide a suitable resection range and predict postoperative seizure outcomes (6). Additionally, characteristic TSC lesions on early brain MRI are correlated with seizure development and neurodevelopmental outcomes during the first 2 years of life (7). Neuroimaging studies have significantly contributed to lesion detection by revealing group-level differences compared with healthy controls; however, their clinical translatability is limited due to insufficient individual-level precision.  Machine learning (ML) algorithms represent highly suitable for individualized medicine due to the fact that they can be trained with vast amounts of data and are able to consider novel data sources, including genetic profiles, imaging, EEG recordings, and physiological data (8, 9). ML models based on brain images can predict epilepsy risk in several neurological disorders, along with classifying epilepsy patients with high accuracy and identifying neuroanatomical features associated with epilepsy, even in non-lesional (MRI-negative) cases (10-13). Radiomics is described as a highly automated computational method that can extract and analyze large amounts of advanced quantitative imaging features (14). Its utility has been demonstrated in predicting postoperative seizure recurrence (15), poststroke cognitive impairment (16), and hematoma expansion after intracerebral hemorrhage (ICH) (17). Although radiomics and ML algorithms have been applied to identify epi foci in intractable epilepsy (18), their potential use for predicting such foci in TSC remains unexplored. | |
| 1. **Objectives** | This research aims to develop and validate a noninvasive, clinically applicable predictive model for epi foci identification and surgical outcome assessment in patients with TSC. | |
| 1. **Study design** | All procedures were performed according to the guidelines of the Declaration of Helsinki of the World Medical Association and the Ethics Committee guidelines of Xinqiao Hospital (approval number: RN202502601), and this study had been registered on Chinese Clinical Trial Registry with the registered number of ChiCTR2500098144. | |
| 1. **Study duration** | All patients who underwent resection surgery were enrolled after they had presented to the three epilepsy centers before October 2023, with at least one year of follow-up in October 2024. | |
| 1. **Study population** | TSC patients (n=144) | |
| 1. **Study procedure** | 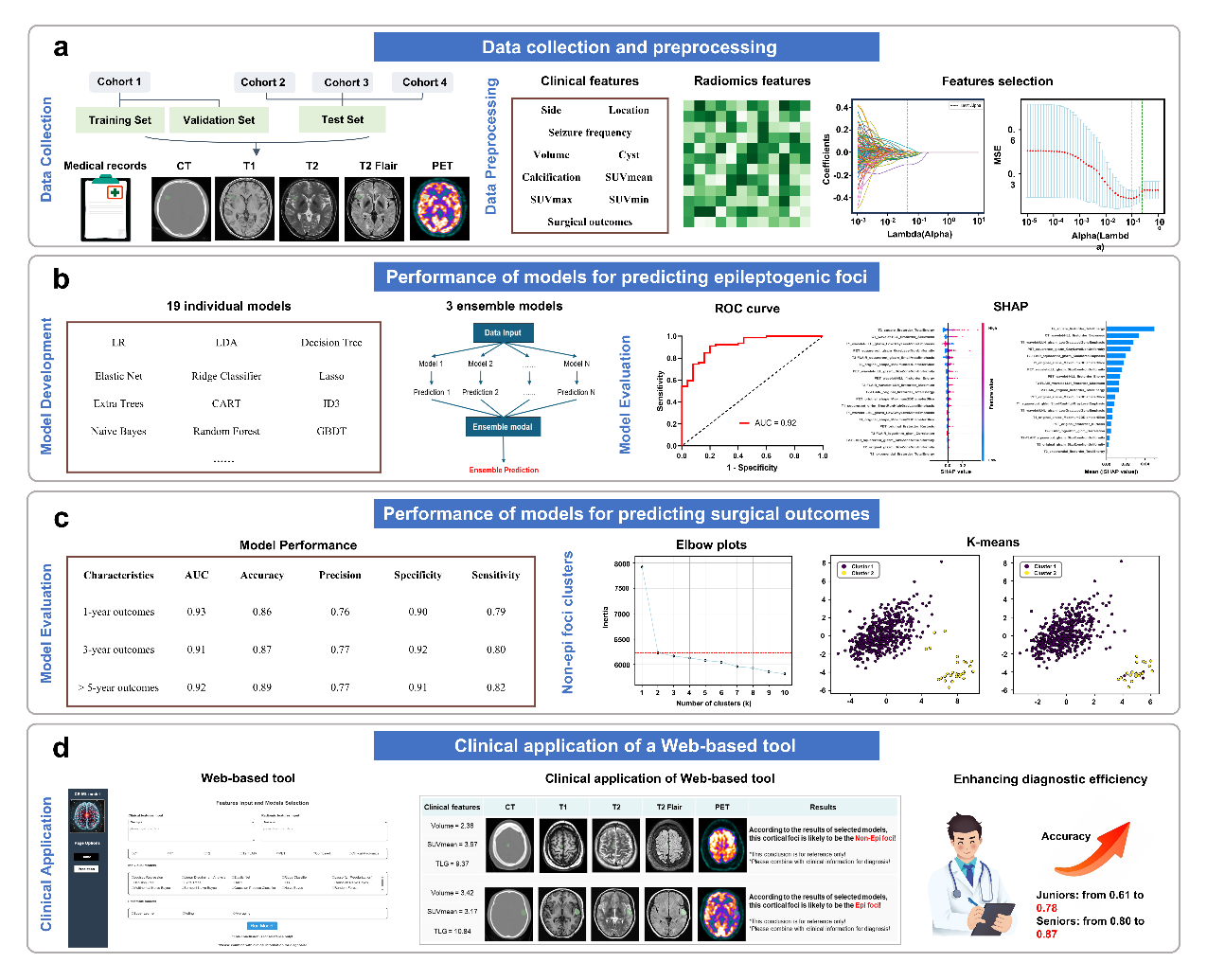Fig 1. The overall procedure of the study. (a) Data collection and preprocessing. (b) Performance of models for predicting epileptogenic foci. (c) Performance of models for predicting surgical outcomes. (d) Clinical application of a Web-based tool. | |
| 1. **Inclusion criteria** | (i) diagnosed with TSC according to the international TSC diagnostic criteria in 2021; (ii) experienced intractable epilepsy and seizures at least twice per month on average during the six months prior to surgery; (iii) underwent comprehensive preoperative evaluations and epilepsy surgery at the comprehensive epilepsy center; and (iv) completed at least 1 year of follow-up. | |
| 1. **Exclusion criteria** | (i) a history of other specific neurological abnormalities, mainly including encephalitis, hydrocephalus, intracerebral hemorrhage or cerebrovascular disease; (ii) contraindications for imaging examinations or incomplete imaging data; and (iii) dropout or loss to follow-up. | |
| 1. **Study endpoints** | Finish at least one year of follow-up in October 2024. | |
| 1. **Adverse events** | This study is not associated with adverse events. | |
| 1. **References** | 1. Curatolo P, Moavero R, de Vries PJ. Neurological and neuropsychiatric aspects of tuberous sclerosis complex. The Lancet Neurology. 2015;14(7):733-45.  2. Northrup H, Aronow ME, Bebin EM, Bissler J, Darling TN, de Vries PJ, et al. Updated International Tuberous Sclerosis Complex Diagnostic Criteria and Surveillance and Management Recommendations. Pediatric neurology. 2021;123:50-66.  3. Liu S, Yu T, Guan Y, Zhang K, Ding P, Chen L, et al. Resective epilepsy surgery in tuberous sclerosis complex: a nationwide multicentre retrospective study from China. Brain. 2020;143(2):570-81.  4. Krsek P, Jahodova A, Kyncl M, Kudr M, Komarek V, Jezdik P, et al. Predictors of seizure-free outcome after epilepsy surgery for pediatric tuberous sclerosis complex. Epilepsia. 2013;54(11):1913-21.  5. Kong Y, Cheng N, Qiu F-j, Yao L, Gao M, Chen A-q, et al. Application value of multimodal MRI combined with PET metabolic parameters in temporal lobe epilepsy with dual pathology. European Journal of Radiology. 2023;169:111171.  6. Walger L, Adler S, Wagstyl K, Henschel L, David B, Borger V, et al. Artificial intelligence for the detection of focal cortical dysplasia: Challenges in translating algorithms into clinical practice. Epilepsia. 2023;64(5):1093-112.  7. Hulshof HM, Kuijf HJ, Kotulska K, Curatolo P, Weschke B, Riney K, et al. Association of Early MRI Characteristics With Subsequent Epilepsy and Neurodevelopmental Outcomes in Children With Tuberous Sclerosis Complex. Neurology. 2022;98(12):e1216-e25.  8. Johnson KB, Wei WQ, Weeraratne D, Frisse ME, Misulis K, Rhee K, et al. Precision Medicine, AI, and the Future of Personalized Health Care. Clinical and Translational Science. 2020;14(1):86-93.  9. Wang HE, Woodman M, Triebkorn P, Lemarechal JD, Jha J, Dollomaja B, et al. Delineating epileptogenic networks using brain imaging data and personalized modeling in drug-resistant epilepsy. Science translational medicine. 2023;15(680):eabp8982.  10. Bernardo D, Kim J, Cornet MC, Numis AL, Scheffler A, Rao VR, et al. Machine learning for forecasting initial seizure onset in neonatal hypoxic-ischemic encephalopathy. Epilepsia. 2025;66(1):89-103.  11. Lindgren E, Shu L, Simaan N, Krzywicka K, de Winter MA, Sánchez van Kammen M, et al. Development and Validation of a Clinical Score to Predict Epilepsy After Cerebral Venous Thrombosis. JAMA neurology. 2024;81(12):1274-83.  12. Gleichgerrcht E, Kaestner E, Hassanzadeh R, Roth RW, Parashos A, Davis KA, et al. Redefining diagnostic lesional status in temporal lobe epilepsy with artificial intelligence. Brain. 2025.  13. Jiang Y, Li W, Li J, Li X, Zhang H, Sima X, et al. Identification of four biotypes in temporal lobe epilepsy via machine learning on brain images. Nat Commun. 2024;15(1):2221.  14. Lambin P, Rios-Velazquez E, Leijenaar R, Carvalho S, van Stiphout RG, Granton P, et al. Radiomics: extracting more information from medical images using advanced feature analysis. European journal of cancer (Oxford, England : 1990). 2012;48(4):441-6.  15. Sun K, Liu Z, Li Y, Wang L, Tang Z, Wang S, et al. Radiomics Analysis of Postoperative Epilepsy Seizures in Low-Grade Gliomas Using Preoperative MR Images. Frontiers in oncology. 2020;10:1096.  16. Betrouni N, Yasmina M, Bombois S, Pétrault M, Dondaine T, Lachaud C, et al. Texture Features of Magnetic Resonance Images: an Early Marker of Post-stroke Cognitive Impairment. Translational stroke research. 2020;11(4):643-52.  17. Ma C, Zhang Y, Niyazi T, Wei J, Guocai G, Liu J, et al. Radiomics for predicting hematoma expansion in patients with hypertensive intraparenchymal hematomas. Eur J Radiol. 2019;115:10-5.  18. Yang S, Chen S, Huang Y, Lu Y, Chen Y, Ye L, et al. Combining MRI radiomics and clinical features for early identification of drug-resistant epilepsy in people with newly diagnosed epilepsy. Epilepsy Behav. 2025;162:110165. | |
